# Supplementary material for: Protein carbonyl determination by a rhodamine B hydrazide-based fluorometric assay
Source: Redox Biol. 2018 Apr 25;17:236–45. doi: 10.1016/j.redox.2018.04.017 (PMC6006725; doi:10.1016/j.redox.2018.04.017)
Supplement: Supplementary file 1 — Supplementary material [file mmc1.docx]

**Protein carbonyl determination by a rhodamine B hydrazide-based fluorometric assay**

**Christos D. Georgiou^1^, Dimitrios Zisimopoulos^1^, Vasiliki Argyropoulou^1^, Electra Kalaitzopoulou^1^, Panayiotis V. Ioannou^2^, George Salachas^3^, Tilman Grune^4^**

^1^Department of Biology, University of Patras, Patras, Greece

^2^Department of Chemistry, University of Patras, Patras, Greece

^3^Department of Agricultural Technology, TEI of Western Greece, Patras, Greece

^4^Department of Molecular Toxicology, German Institute of Human Nutrition, Nuthetal, Germany

DZ, VA and EK contributed equally

**Corresponding author:** Christos D. Georgiou,

E-mail: c.georgiou@upatras.gr

**Abstract**

A new fluorometric assay is presented for the ultrasensitive quantification of total protein carbonyls, and is based on their specific reaction with rhodamine B hydrazide (RBH), and the production of a protein carbonyl-RBH hydrazone the fluorescence of which (at ex/em 560/585 nm) is greatly enhanced by guanidine-HCl. Compared to the fluorescein-5-thiosemicarbazide (FTC)-based fluorometric assay, the RBH assay uses a 24-fold shorter reaction incubation time (1 hr) and at least 1000-fold lower protein quantity (2.5 µg), and produces very reliable data that were verified by extensive standardization experiments. The protein carbonyl group detection sensitivity limit of the RBH assay, based on its standard curve, can be as low as 0.6 pmol, and even lower. Counting the very low protein limit of the RBH assay, its cumulative and functional sensitivity is 8,500- and 800-fold higher than the corresponding ones for the FTC assay. Neither heme proteins hemoglobin and cytochrome *c* nor DNA interfere with the RBH assay.

**Keywords:** fluorometric method; rhodamine B hydrazide; protein carbonyls; protein oxidation; oxidative stress; DNA

**Abbreviations**

Rhodamine B hydrazide (RBH)

Fluorescein-5-thiosemicarbazide (FTC)

Fluorescence specific activity (FSA)

**Introduction**

The biological importance of protein carbonylation as a marker of oxidative stress, the mechanisms of protein carbonyl formation, the involvement of carbonylated proteins in various biological phenomena and diseases, and their partial recognition/decomposition by proteasomes have been extensively discussed in a previous study **[1]**. In the same study, the numerous 2,4-dinitrophenylhydrazine (DNPH)-based assays for protein carbonyl identification (quantification in total protein samples or their qualitative spatial distribution in cells) have been also examined, together with their limitations and other reliability problems (due to non-standardized protein fractionation protocols, DNA and sulfenic acid interference among other factors).

There are also numerous non-DNPH-based methods for the quantitation of protein carbonyls by non-fluorescent and fluorescent hydrazides. Indicatively, the non-fluorescent sodium cyanoborohydride has been coupled with fluoresceinamine (FINH_2_) for the identification of the FINH_2_-carbonyl derivative by HPLC and mass spectrometry (MS) **[2]**. However, the resulting FINH_2_ derivatives are degraded by acid hydrolysis to non-fluorescent decarboxylated derivatives **[3]**. Alternatively, the non-fluorescent biotin hydrazide **[4, 5]** can track carbonylated proteins via fractionation techniques (e.g., microcapillary LC) and their specific identification by MS **[6]**. Moreover, biotin hydrazide-labeled total proteins can be detected by streptavidin-coupled peroxidase-catalyzed chemiluminescence of immunoblots **[7]**.

On the other hand, fluorescent hydrazides have been mostly used for qualitative protein carbonyl assessment. For example, coumarin hydrazide (which also fluoresces in free form) has been used in microscopy imaging of cellular protein (and lipid) carbonyls **[8]**. Biotin hydrazide-labeled total proteins are semi quantitatively evaluated by streptavidin-coupled fluorescence in Western immunoblots **[9]**. This coupling is also combined by use of SDS **[10, 11]** and carbonyl reduction with tritiated (^3^H-labeled) sodium borohydride **[12]**. Sensitivity and non-reliance on antibodies (which are prone to non-specific background noise and contamination of endogenous immunoglobulins) are the main advantages of the biotin hydrazide-labeling techniques. Fluorescent Bodipy, Cy3 and Cy5 hydrazide (they also fluoresce in their free forms) have been used to semi quantitatively detect derivatized total proteins fractionated in a two-dimensional gel, which overcomes the need of Western blotting and immunodetection, thereby shortening procedure time and increasing accuracy **[13]**. Other methods use the fluorescent (also in free form) probes fluorescein hydrazide **[14]** and fluorescein thiosemicarbazide (FTC) **[14-17]** for the fluorometric determination of total protein carbonyl groups after fractionation on PAGE. However, the aforementioned fluorescent methods have not been developed for the routine quantification of the carbonyl content in total protein samples. Currently, this need is served by a method based on fluorescein-5-thiosemicarbazide (FTC) **[14, 15, 17]**. However, the FTC assay (has been offered as a commercial kit) has the following limitations, which necessitate the development of a more reliable fluorescent assay.

**The fluorometric FTC assay: limitations**

To illustrate the limitations of the FTC assay a briefly outline of the principle of this assay is needed: Protein carbonyls are incubated for 24 hrs (or overnight) with 0.2 mM FTC at pH 6.0, followed by removal of the unreacted (free) fluorescent FTC by 16%-TCA protein precipitation, and 3x-wash of the resulting protein pellet with acetone **[17]**. The main limitations of the FTC assay are as follows:

1. The protein precipitation step by TCA alone has been shown that is quite ineffective for protein recovery (e.g., recovery can be as low as 24% **[18]**), and also results in loss of the acid soluble proteins.
2. The assay is applicable only to samples with high in protein content, e.g. on 5 mg ml^-1^ for human plasma and up to 10 mg ml^-1^ protein **[17]**.
3. The FTC assay loses further in sensitivity because the TCA-precipitated and 3x-acetone-washed protein must be solubilized in 6 M gndHCl, which quenches the fluorescence of the protein carbonyl-FTC hydrazone, and to overcome this the resulting hydrazone solution must be 10-fold diluted. The dilution step is also required to minimize interference in the accurate determination of the solubilized protein concentration by the employed BCA assay **[17]**.
4. Another crucial uncertainty of the FTC assay is that although it uses a 1:1 fluorescent molar stoichiometry between the protein carbonyl-FTC hydrazone and the FTC reagent used for the standard curve **[17]**) claimed to be based on a previous study **[14]**, the existence of such stoichiometry is not reported by the referred study.
5. The question of DNA interference has not been addressed for the FTC assay.

The aforementioned limitations of the FTC assay illustrate the need for the development of a new fluorometric assay that is simple, fast, reproducible, and highly sensitive both in the routine analysis of total protein samples at the µg level and with very low carbonyl content. A standardized methodology for sample protein fractionation is reported elsewhere **[1]**.

The present study addresses the aforementioned limitations of the FTC assay by developing a new fluorometric assay based on rhodamine B hydrazide (RBH). It is based on the reaction of RBH with isolated protein samples (at pH 3), their subsequent precipitation with a combination of deoxycholate (DOC) and trichloroacetic acid (TCA), the solubilization of the protein carbonyl-RBH hydrazone in near saturation guanidine hydrochloride (gndHC) at pH 5, and its subsequent fluorescence quantification (at ex/em 560/585 nm).

RBH has never been used for the quantification of protein carbonyls, except as a tag in reactive carbonyl containing short peptides (fused to T4 lysozyme or synthesized on filter paper for colorimetric assays of the peptide-hydrazide interaction), used for site-specific protein labeling **[19]**). The only time RBH has been used to quantify a hydrazone derivative was with diacetyl (by formation of a hydrazone bond with one of its two carbonyl groups **[20]**); also for the quantification of malondialdehyde in biological fluids (via derivatization of its aldehyde group, and HPLC quantification **[21]**). Other than these cases, RBH has been used for the quantification of the following molecules via their direct/indirect oxidation and decompose of RBH to its fluorescent product rhodamine B: **peroxynitrite** (via direct oxidation of RBH **[22]**; **H_2_O_2_** (via iron-tetrasulfonatophthalocyanine catalysis), and also biomolecules that produce H_2_O_2_ via catalysis by their oxidase (such as **glucose** via glucose oxidase) **[23]**; **Cu^2+^** (by its binding to the spirolactam amide ring of RBH, thereby hydrolyzing it to rhodamine B **[24]**); **NO** indirectly (it converts NO_2_ aerobically to N_2_O_3_, which, via nitrosylation, converts the hydrazide amino group of RBH to diazonium group, the 5-membered ring of which is opened to form an azide intermediate that is converted into rhodamine B) **[25]**; **hemoglobin and cytochrome *c*** (via RBH hydrolysis to rhodamine B after RBH incorporation in detergent sodium dodecylbenzene sulfonate micelles **[26, 27]**).

However, the aforementioned RBH oxidants are not expected to pose any interference problems with the RBH assay, (i) because the assay is not applied on whole homogenates but only on isolated protein fractions (possibly coexisting with hemoglobin and cytochrome *c*, the interference of which, and of DNA, will be investigated), and (ii) these oxidant molecules are not bound to proteins (not even the readily reacting with proteins peroxinitrite **[28]**). Nonetheless, ROS-oxidized proteins may contain organic hydroperoxides (PrOOH **[29]**) with oxidative properties similar to H_2_O_2_, and could oxidize RBH to rhodamine B. However, PrOOH will be destroyed by dithiothreitol (DTT) when pre-treating samples by a standardized protein fractionation procedure suggested for quantifying protein carbonyls (described in supplement Section V of the ntrDNPH assay **[1]**); DTT reduces organic hydroperoxides (e.g. of lipids) to alcohols **[30]**, and also eliminates thydrazine reactive sulfenic groups in ROS-oxidized Cys residues. However, even using non-DTT-treated protein samples that oxidize RBH to rhodamine B in the reaction mixture of the RBH assay, rhodamine B will not interfere because it is removed from the RBH-treated protein by DOC-TCA-precipitation of the latter (and its subsequent solubilization for the quantification of the protein carbonyl-RBH hydrazone).

Nonetheless, the RBH assay will be extensively standardized in order to address possible unreliability and interference and considerations. Besides suggesting use of the aforementioned standardized protein fractionation procedure, which also removes DNA **[1]**, the following standardization studies will be performed: 1. RBH assay parameters (pH, reaction time, fluorescence maximization and stability of the protein carbonyl-RBH hydrazone) will be developed on control proteins (BSA, lysozyme, pepsin) that will be artificially carbonylated (by Fenton-generated hydroxyl radical oxidation, ox) and decarbonylated (by NaBH_4_ reduction, red); the RBH assay will be cross-checked on the control protein pairs BSA_ox/red_, lysozyme_ox/red_, and pepsin_ox/red_. 2. Two-fold titration experiments will be performed on the RBH assay to establish the molar stoichiometry of the reaction between RBH and protein carbonyl groups (thereby allowing the investigation of using RBH for the assay’s standard curve): (i) titration of a known concentration of RBH by known concentrations of protein carbonyls (from control BSA_ox_, determined by the control ntrDNPH assay **[1]**); (ii) titration of a known concentration of BSA_ox_ carbonyls by known concentrations of RBH. 3. Statistical data coincidence comparisons will be performed between the RBH assay and the control ntrDNPH assay, using known carbonyl concentrations of BSA_ox_ mixed at known ratios with BSA_red_ (decarbonylated control). The ntrDNPH assay is used as comparison control because (i) the reaction mechanism of protein carbonyls with DNPH is well known, and (ii) the reliability in measuring them with DNPH has been maximized with the ntrDNPH assay **[1]**. 4. The carbonyl content of indicative protein samples will be tested with the RBH assay against their artificially decarbonylated (via NaBH_4_ reduction) counterparts, and will be compared with that determined by the control ntrDNPH assay. 5. Finally, the RBH assay will be tested for interference by DNA (in case samples are not treated by the aforementioned standardized protein fractionation procedure), hemoglobin, and cytochrome *c*.

**Materials and Methods**

**Reagents**

Acetone (AC; Merck, cat. no. 01-6300117)

Ammonium iron (II) sulfate hexahydrate (NH_4_)_2_Fe(SO_4_)_2_•6H_2_O; Sigma, cat. no. 215406)

Βutylated hydroxyanisol (BHA; Sigma-Aldrich, cat. no. B1253)

Bovine serum albumin (BSA; Sigma, cat. no. A9418)

Chloroform (CHCl_3_; Merck, cat. no. 1.02445)

Citric acid monohydrate (Sigma, cat no. C1909)

Coommasie Briliant Blue G-250 (CBB G-250; Serva, cat. no. C.I. 42655)

Cytochrome *c* from equine heart (cyt.*c*; Sigma, cat. no. C2506)

Deoxycholic acid, sodium salt (DOC; Sigma-Aldrich, cat. no. D6750)

2,4-Dinitrophenylhydrazine (DNPH; Sigma, cat. no. D198501)

DNA type III from salmon testes (Sigma, cat. no D1626)

Ethanol, absolute (EtOH; Merck cat. no. 159010)

Ethylenediaminetetraacetic acid disodium (EDTA; Merck, cat. no. 324503)

Guanidine-HCl (gndHCl; Sigma, cat. no. G4505)

Hydrazine hydrate (Sigma, cat. no. 225819)

Hydrochloric acid (HCl, ≥37% w/w; Fluka, cat. no. 84415)

Hemoglobin from equine (Hb; Sigma, cat. no. H4632)

Hydrogen peroxide (H_2_O_2_; 30% w/w, Merck, cat. no. 107209)

Lysozyme from chicken egg white (Sigma, cat no. L6876)

Methanol (MetOH; 100%) for HPLC (Sigma-Aldrich, cat. no. 34860)

Pepsin from porcine gastric mucosa (Sigma, cat no. P6887)

Rhodamine B (Sigma, cat. no. R6626)

Sodium borohydride (NaBH_4_; Sigma, cat. no. 213462)

Sodium chloride (NaCl; Sigma, cat. no. 433209)

Sodium hydroxide (NaOH; Merck, cat. no. 567530)

Sodium (di-) phosphate (Νa_2_HPO_4_•2H_2_O; Merck, cat. no. 106580)

Sodium (tri-) phosphate dodecahydrate (Νa_3_PO_4_•12H_2_O; Merck, cat. no. 106578)

Trichloroacetic acid (TCA; Merck, cat. no. 1.00807.0250)

Urea (Sigma-Aldrich, cat. no. U1250)

Rhodamine B hydrazide (RBH): RBH was synthesized according to a published procedure **[20, 22]**. The product was recrystallized from methanol-acetone as a colorless solid with purity >98% by TLC (yield: ~0.28 g), and identified by HPLC-MS. RBH is supplied by Synchem UG & Co. KG, Germany (order number: am005; http://www.synchem.de/chemical_Rhodamine%20B%20hydrazide.html),

Hangzhou Sage Chemical Co., Ltd. China (cat # SGC29286) as Spiro[1H-isoindole-1,9'-[9H]xanthen]-3(2H)-one,2-amino-3',6'-bis(diethylamino)-(http://sagechem.lookchem.com/products/CasNo-74317-53-6-Spiro-1H-isoindole-1-9---9H-xanthen--3-2H--one-2-amino-3-6--bis-diethylamino---11049789.html), and Cayman Chemical (item no 23133; https://www.caymanchem.com/product/23133). RBH has been previously offered by Sigma-Aldrich (prod. no 83684) but it is currently discontinued.

All other reagents used were of the highest purity.

***Instrumentation***

Balance (Kern, 770/65/6J)

Bench top centrifuge (Hermle, model Z206A)

Glass Pasteur pipettes (internal diameter 0.5 cm, 22 cm long, by Hirschmann Laborgeräte GmbH & Co, Germany)

Microcentrifuge clear tubes, 1.5 and 2 ml (VWR, cat. no. 89000-028)

Micropipettes (adjustable volume) 2.5 μl, 10 μl, 20 μl, 100 μl, 200 μl, 1 ml, and tips (Eppendorf Research)

Microcuvette for absorbance measurements (12.5×12.5×45 mm external dimensions, 4 mm internal window and 9 mm bottom, 1.16 ml, quartz; Starna 9/B/9/Q/10)

Microcuvette for fluorescence measurements (45×4 mm, 0.5 ml, quartz; Starna SOG/Q), fitted in a Starna FCA4 adapter

pH meter (Metrohm, 827 pHlab)

Speedvac apparatus for vacuum drying

Spectrofluorometer (Shimadzu, model RF-1501)

Spectrophotometer (Hitachi, model UV-VIS U-1800)

Dialysis membrane (Spectrum Laboratories Inc Spectra/Por 4- Dialysis Membrane Tubing, MWCO 12-14,000)

**Part A. Protocol of the RBH assay**

The following RBH assay procedure has been developed after standardization of its following parameters: assay reaction pH (previously studied for the reaction of RBH with diacetyl’s carbonyls **[20]**); assay reaction time, optimum pH and excitation/emission (ex/em) wavelengths for attaining maximum fluorescence (units, FU) of the protein carbonyl-RBH hydrazone, all in the presence of chaotropic factors (gndHCl and urea) in order to minimize reaction time and maximize hydrazone FU and stability over time; establishment of the molar stoichiometry of the reaction between RBH and protein carbonyl groups, which is 1:1 and allows the use of RBH for constructing the standard curve of the RBH assay. Standardization experiments were performed on certain control protein (BSA_ox/red_, lysozyme_ox/red_, pepsin_ox/red_), and are presented in the Supplement’s *Part I. Standardization of the RBH assay, Sections I*, *II*, *III*, *IV*. Having established that the RBH assay (see mechanism in **Fig. 1**) will be performed at 25 µM RBH in 33/17 mM C/P - 0.1 M gndHCl buffer, pH 3, for 1 hr at 37^o^C in the dark, using 33/17 mM C/P - 8 M gndHCl buffer, pH 5, as solubilizing solution of the DOC-TCA-precipitated/cold acetone-washed protein carbonyl-RBH hydrazone, RBH assay optimized procedure and reagent solutions are as follows:

- **132/68 mM C/P - 0.4 M gndHCl buffer, pH 3:** Dissolve 0.118 g K_2_HPO_4_, 0.277 g citric acid monohydrate and 0.382 g gndHCl in ~9 ml ddH_2_O to final 10 ml, and adjust to pH 3 (with 10 M KOH dropwise).

**1 mM RBH**: Dissolve 1 mg RBH in 2 ml absolute EtOH. The RBH stock solution should be always kept light-protected (e.g. wrapped in aluminum foil) and placed in an ice-water bath during use both in the RBH assay and for preparing the RBH standard curve (in the supplement’s *Part I. Standardization of the RBH assay, Section IV*). It can be stored at -20^o^C for at least a month.

- **1.4 M HCl:** Mix 0.117 ml conc. HCl (12 M) with 0.875 ml ddH_2_O.
- **100% TCA:** For the 100% stock, dissolve 10 g TCA in ddH_2_O to final 10 ml.
- **1% DOC**: Dissolve 0.01 g DOC in ddH_2_O to final 1 ml.
- **100% Acetone**: Keep at -20°C.
- **33/17 mM C/P - 8 M gndHCl buffer, pH 5**: Dissolve 30 mg K_2_HPO_4_, 69 mg citric acid monohydrate and 7.64 g gndHCl in ~4 ml ddH_2_O to final 10 ml (by heating the solution to 35°C for approximately 30 min), and after cooling to RT adjust to pH 5 (with KOH).

***Sample protein preparation and solubilization***: Proteins in biological samples can be isolated as *cytoplasmic/aqueous*, *membrane/lipid-bound* or *histone/DNA-bound* protein fractions by the aforementioned standardized procedure (see supplement’s *Section IV* of the ntrDNPH assay **[1]**). The blood serum, cytoplasmic and the *histone protein* fraction pellet (isolated from indicative samples tested in the present study; see **Table 3**) are solubilized (e.g. by a glass rod, combined with vortexing, in a microcentrifuge tube by) in a minimum volume of 50 mM NaOH, and used immediately (or kept frozen at -20^o^C; it can be stored for at least one month). The same alkaline solvent is also used for further dilution of the (in minimum volume) protein solubilizate, keeping in mind that the RBH assay requires extremely small amounts of protein (its minimum protein limit is ~2.5 µg).

*IMPORTANT NOTES: 1. Minimum volume solubilizate of large size protein pellets may become gel-like when re-thawed after storage at -20^o^C, which may require an additional minimum dilution with 50 mM NaOH for complete re-solubilization. 2. Sample protein solubilizate pH should be kept at ≤12, since above it protein S-S bonds are unstable at 25ºC (at pH ~13, i.e., at 0.2 M NaOH* ***[31]****).*

***Procedure (timing 90 min)***

**1.** In each of two 1.5 ml-microcentrifuge tubes (one for sample, S, and one for sample blank, SB) mix 50 μl 132/68 mM C/P - 0.4 M gndHCl buffer, pH 3, with 140 μl of an appropriate dilution (with 50 mM NaOH) of the sample protein solution. For highest accuracy prepare S and SS in three dilutions at 2.5 to 50 µg protein. Then to both tubes add 5 μl 1.4 M HCl, and then add 5 μl 1 mM RBH in the S tube, and 5 μl absolute EtOH in the SB tube, and incubate in the dark for 1 hr at 37^o^C (until maximum formation of the protein carbonyl-RBH hydrazone). If sample protein solubilizate is of limited quantity, SB can be prepared only for the lowest dilution, and its netFU_SB_ (see **step 4**) can be proportionally extended as controls to the other S dilutions.

The simplest way to separate (and discard) unreacted RBH from the protein carbonyl-RBH hydrazone and collect the entire protein in the S and SB reaction mixtures is by ultrafiltration using commercially available microcentrifugal filter devices (such as the Amicon Ultra-0.5 ml 10K and the Centricon Ultracel YM-10 by Millipore, with 10K denoting that the filter of this particular microcentrifugal device type has a 10,000-nominal molecular weight limit). This procedure is described in the following **step 1.1**. In the absence of such ultrafilter device the procedure continues in **step 2**.

**1.1.** Place the contents of the S and the SB tube in separate microcentrifugal filter devices (e.g. Amicon Ultra-0.5 ml 10K), centrifuge (at 15,000 g for 15 min at 4ºC) and wash the retained protein (from the unreacted RBH and the other assay reagents) 2x with 0.5 ml ddH_2_O and 1x with 0.5 ml 3/17 mM C/P - 8 M gndHCl buffer, pH 5 (each wash followed by centrifugation). Then, solubilize unreacted RBH-washed protein (that is retained on the filter) in the S and SB microcentrifugal filter device protein with 300 μl (or higher, depending of the volume of the quartz cuvette in use) 33/17 mM C/P - 8 M gndHCl buffer, pH 5, and collect the S and SB solubilizates by centrifugation after reversing the position of the filter device in its microcentrifuge tube holder (see user guide for more details). Since this and similar devices recover the protein with >95% efficiency there is no need to re-determine its concentration in the solubilizates. Then, measure the FU of the S against the FU of the SB solubilizate, and convert to pmol carbonyls μg^-1^ protein as described in the subsequent **step 4**.

**2.** After 1 hr incubation in **step 1**, add to both tubes 4 μl 1% DOC (final 0.02% in the 0.2 ml assay reaction mixture) and incubate for 10 min at RT. Then, add 22 μl 100% TCA (final 10%; for histones see the following *IMPORTANT NOTE 1*) and incubate for 15 min in an ice-water bath. Precipitate proteins in the cloudy solution by centrifugation at 16,000 *g* for 5 min at 4ºC, wash the resulting protein pellet 2x with 0.5 ml cold 100% acetone (see following *IMPORTANT NOTE 2*) each time, followed by centrifugation at 16,000 *g* for 5 min at 4ºC, and dry the pellet (in a speedvac apparatus, or over a steam of air or nitrogen gas) for 10 min at RT. At this point, the S pellet contains the protein carbonyl-RBH hydrazone, and the SB pellet any interfering components present also in the S pellet (for protein pellet storage see following *IMPORTANT NOTE 3*).

*IMPORTANT NOTES: 1. Since histones are acid-soluble proteins, TCA in* ***step 2*** *needs to be set at final 33% (by addition of 0.1 ml 100% TCA to the 0.204 ml mix). 2. When vortexing with 0.5 ml acetone, part of the protein pellet may spread as a thin layer on the internal wall of the centrifuge tube (and above its bottom where the maim pellet is precipitated), which may not be visible if the pellet is small. Thus, while vortexing, scrap this invisible film downwards the internal walls by the round tip of a metallic spatula, and caution should be taken to wet carefully this area with the buffer used to solubilize the pellet described in* ***step 3****. 3. Both the S and SB protein pellets can be stored almost indefinitely at -25^o^C, because the FU of any RBH released from a possible hydrolysis of the protein carbonyl-RBH hydrazone bond in the dry protein pellets will equal to its FU while bound to the protein carbonyl when the pellets will be solubilized and measured as in* ***step 4****.*

**3.** Solubilize the DOC-TCA precipitated/acetone-washed S and SB protein pellets from **step 2** in 50 μl 33/17 mM C/P - 8 M gndHCl buffer, pH 5, by repeated wetting of the internal sides above the 0.5 ml level (by pumping/emptying the 50 μl with the pipette, followed by vigorous vortexing) of the microcentrifuge tube (see *IMPORTANT NOTE 2* in **step 2**), and collect the 50 μl solubilizate at the bottom of the tube by a brief centrifugation. Then, withdraw 10 μl of the 50 μl solubilizate (the 20%) and mix with 90 μl ddH_2_O. This will dilute the 8 M gndHCl component of the 33/17 mM C/P - 8 M gndHCl buffer (pH 5) by 10 fold (further dilution is also done with ddH_2_O) for protein determination (as stated elsewhere **[32]**), using as protein assay reagent blank a mixture of 45 μl ddH_2_O and 5 μl 33/17 mM C/P - 8 M gndHCl buffer, pH 5.

*IMPORTANT NOTES: 1. GndHCl up to 2 M does not interfere with the protein assay* ***[32]*** *used in the present study. 2. The aforementioned protein assay detects minimum 100 ng protein in a sample volume 50 μl mixed with 950 μl of the CBB reagent. Therefore, the initial 50 μl S and SD solubilizate should contain each minimum 1 µg protein (thus the RBH assay minimum quantity required is 2-2.5 µg), in order for the withdrawn 10 μl to contain 0.2 µg protein in their finally adjusted volume of 100 µl, or the minimum 100 ng detected protein in the 50 μl sample volume used by the protein assay. Similar protein concentration should be followed for any other protein assay selected for application with the RBH assay.*

**4.** Adjust the volume of the remaining 40 μl of the S and SB protein pellet solubilizates (from the initial 50 μl solubilizates from **step 3**) to 300 μl (or higher, depending of the volume of the quartz cuvette in use) by addition of 260 μl 33/17 mM C/P - 8 M gndHCl buffer, pH 5 (using the same buffer if further dilution of this solubilizate is needed). Centrifuge the resulting solubilizates at 16,000 *g* for 5 min at 25ºC to precipitate any present DNA (see *IMPORTANT NOTE 1*), and collect carefully the S and SB clear supernatant solubilizates by placing the pipette tip on the opposite (to the centrifugal force direction) bottom side of the tube as to not disturb the DNA clear pellet. Subsequently, determine the net FU of the S and SB solubilizates (designated netFU_S_ and netFU_SB_, respectively) by measuring their FU at ex/em 560/585 nm (with the spectrofluorometer Shimadzu RF-1501 set at 10 nm width slit and high sensitivity) and subtracting from each the FU value of the 33/17 mM C/P - 8 M gndHCl buffer, pH 5.

*IMPORTANT NOTES: 1. Any DNA present in the S and SB is not solubilized in the 33/17 mM* *C/P - 8 M gndHCl buffer, pH 5 (this is shown in supplement’s Part II. Test of DNA interference on the RBH assay), and forms a clear viscous pellet at the bottom of the centrifuge tube. Centrifugation should be performed at 25ºC because the highly concentrated gndHCl solution forms gndHCl crystals at low temperature. 2. Fluorescence measurements among different samples should be made after having washed the cuvette sequentially with ddH_2_O (to remove any gndHCl remnants deposited in the internal walls of cuvette from the previous sample), 100% MetOH (to remove any free RBH/RBH hydrazone remnants), ddH_2_O, and finally with the 33/17 mM* *C/P - 8 M gndHCl buffer. Washing the cuvette only with the 33/17 mM* *C/P - 8 M gndHCl buffer may be sufficient when measuring increasing fold dilutions of the same sample as those described in the subsequent IMPORTANT NOTE 4. 3. To ensure that RBH (at 25 µM) is in excess compared to the unknown protein carbonyls during reaction, at least 3 proportional dilutions (containing 2.5 to 50 µg) of the unknown sample protein solution should be tested for achieving correspondingly proportional FU values. 4. If the FU value of the S solubilizate exceeds the FU scale of the spectrofluorometer in use, it is appropriately diluted with 33/17 mM* *C/P - 8 M gndHCl buffer, pH 5, and its FU is re-measured. 5. The molar fluorescence of the RBH assay is stable for at least 2 days in the dark at RT.*

Convert the resulting netFU_S_ and netFU_SB_ values per protein quantity (designated *pq*; fully designated netFU_S/pq_ and netFU_SB/pq_, respectively) by dividing them with the protein quantity contained in the corresponding 40 μl S- and SB-pellet solubilizates (and determined as in **step 3**). Then, the net FU of the protein carbonyl (pc)-RBH hydrazone per *pc* in the S solubilizate (designated netS_pc/pq_) is given by the formula:

netS_pc/pq_ = netFU_S/pq_ - netFU_SB/pq_

The netS_pc/pq_ (determined for and being proportional to at least three sample dilutions) is calculated from the netFU_S/pq_ - netFU_SB/pq_ difference after converting the netFU_S_ and netFU_SB_ values to carbonyl pmoles (and dividing them by the corresponding protein quantity, e.g. in μg, determined as in **step 3**) by the RBH standard curve; its construction is described in the supplement’s *Part I. Standardization of the RBH assay, Section IV*), which is made with the same solution of 33/17 mM C/P - 8 M gndHCl buffer (pH 5) that is used throughout the procedure.

*CAUTIONS: 1. The netFU_S/pq_ and netFU_SB/pq_ should be determined separately because the quantity of their precipitated protein in* ***step 2*** *may differ (although they start with same protein quantity). 2. Given that protein carbony-RBH hydrazone fluorescence is measured in the 33/17 mM* *C/P - 8 M gndHCl buffer (pH 5) where gndHCl is at near maximum solubility, for achieving repeatability in the concentration of gndHCl in this buffer, the commercial gndHCl reagent in use should be kept desiccated because it is highly hygroscopic.*

*IMPORTANT NOTES: 1. The highest possible sensitivity of the RBH assay is attained by standardizing the 33/17 mM* *C/P buffer (pH 5) at gndHCl saturation (satur) concentration, and when the background fluorescence of the 33/17 mM* *C/P - gndHCl_satur_ (or at 8 M gndHCl) buffer (pH 5) is at minimum level attained using assay reagents of the highest purity. Given that saturation is temperature dependent, the 33/17 mM* *C/P - gndHCl_satur_ buffer (pH 5) should be kept at constant temperature (e.g. in a water bath set at 25°C). 2. The difference of the factors netFU_S/pq_ and netFU_SB/pq_ in the equation for netS_pc/pq_ should be calculated for various sample dilutions (at least three) to which the corresponding differences should be proportional among each other. If this is not possible (especially for samples of limited quantity and low carbonyl content), the individual values for each of the two equation factors may be plotted separately (against the corresponding dilutions) as to fit to a straight line that crosses both axes at zero value, and the slope values are determined. Then, the equation of netS_pc/pq_ will be equal to the slope value of the factor netFU_S/pq_ plot minus the slope value of the factor netFU_SB/pq_ plot.*

***Statistical treatment of raw data and assay statistical precision*:** The RBH assay was tested on protein fractions from various sources (**Tables 2, 3**). Protein carbonyl content is expressed as mean (of at least 5 independent experiments) ± standard deviation (SD), and statistically analyzed by the package SPSS Inc, 2001, Release 11.0.0, USA. Carbonyl values from human serum, in particular, are mean of 10/10 male/female human subjects in middle age ± SD, after checking for equality of error variances between values of males and females (Levene's test) with two-way ANOVA analysis of variance (to identify significant differences between values), and with the parametric post-hoc multiple comparison Bonferroni test (p <0.05).

The RBH assay is statistically analyzed for precision assessed both during a single analytical run (*within-run*, *within-day precision* or *repeatability*) and with time (*between-run, between day repeatability,* also named *intermediate* *precision*). For determining the minimum statistical variation of the protein carbonyls quantified by the RBH assay, at least three successive dilutions of human serum samples are analyzed the same day of blood collection, and their mean value is calculated. The within-day % coefficient variation is calculated as SDx100/mean, and the variance of intermediate precision (σ^2^_total_) is defined as the sum of between day variance (σ^2^_between_) that is associated with the day-to-day variation, and the variance of repeatability (σ^2^_within_).

**Part B. Standardization of the RBH assay by statistical comparison with the control ntrDNPH assay for data coincidence**

The RBH assay is compared for data coincidence against the control ntrDNPH assay **[1]** for two reasons: (i) the reaction mechanism of protein carbonyls with DNPH has been established after numerous studies; the reliability of measuring protein carbonyls with DNPH has been established by the development of the ntrDNPH assay: Protein carbonyls are measured by the two assays in a series of BSA_ox_, lysozyme_ox_ and pepsin_ox_ samples mixed at various proportions with their corresponding protein_red_ pairs (depleted of protein carbonyls), while keeping constant the total protein concentration. Specifically, the two assays are used to measure the protein carbonyls of increasing quantities of e.g. BSA_ox_ in mixture with decreasing quantities of BSA_red_ (that is, at various BSA_ox/red_ ratios, each tested in triplicates) and at a fixed total protein mix. These mean values are then used to determine the Pearson correlation of these values, and to perform a Bland-Altman plot comparison analysis between the assays (**Fig. 2**), using Prism 6 by GraphPad Software.

**Part C. Standardization of the RBH assay against possible interfering factors**

***Heme proteins*:** As already mentioned in Introduction, RBH has been used for the quantification of Hb and cyt.*c* which hydrolyze it to rhodamine B **[26, 27]**. Since this study does not elucidate the mechanism of RBH reaction with Hb and cyt.*c*, we investigated a possible interference by these heme proteins on the RBH assay using as comparison control the ntrDNPH assay **[1]**. Possible protein heme interference on the RBH assay was tested (against the ntrDNPH) on untreated (untr) preparations of Hb and cyt.*c* (designated Hb_untr_ and cyt.*c*_untr_, respectively), using decarbonylated (NaBH_4_-reduced) Hb_red_ and cyt.*c*_red_ as additional control.Protein carbonyls were determined on 50 to 150 µg heme proteins (from 2-mg ml^-1^ stock solutions, by dilution of their 10-mg ml^-1^ initial stocks; their preparation is shown in the supplement’s *Part I. Standardization of the RBH assay,* *Section I*), against their untreated preparations of same protein quantities (**Table 2**).

***DNA*:** DNA has been claimed to interfer with the 2,4-dinitrophenylhydrazine (DNPH)-based photometric assays without elucidation of the interference mechanism **[33-35]**. However, it was found that this interference is not due to DNPH’s direct reaction with the carbonyl groups in the T, C and G bases of DNA but to its non-specific binding to DNA **[1]**. Since RBH contains also an active hydrazine group, we investigated the possible interference of the RBH assay with DNA (**Table 2**), The detailed experimental procedure is shown in the supplement’s *Part II*. *Test of DNA interference on the RBH assay*.

**Part D. Calculations and expected results on indicative samples**

***RBH assay optimal standardization parameters***: The shortest reaction time (1 hr) for protein carbonyl-RBH hydrazone adduct formation was obtained in the presence of the chaotropic reagent gndHCl (**Suppl.** **Fig. 1**), but only at up to 0.1 M. GndHCl at 0.1 M is high enough to induce an effective access of RBH to protein carbonyls due to the partial unfolding it causes to proteins **[36]**, but low enough as to not prevent (via its H-bond disrupting action) the electrostatic association of RBH with proteins and its subsequent reaction with their carbonyls. Another important finding of this study is that the net fluorescence units (FU) of the BSA_red_ (and also the pepsin_red_ and lysozyme_red_) control (corrected for the fluorescence of the sample blank) are zero, and similarly the FU of the reagent blank, which shows that the 2x acetone wash procedure in the RBH assay completely eliminates any RBH non-specific binding to proteins.

The maximum fluorescence specific activity (FSA) of the protein carbonyl-RBH hydrazone adduct takes place at pH 5 as a function of gndHCl concentration. It reaches a ~130-fold increase at 8 M gndHCl (**Suppl.** **Fig. 2**), and it further increases by at least 3-fold at saturated gndHCl concentration (data not shown). It should be noted that the pH profile of the maximum FSA value of the protein carbonyl-RBH hydrazone in the absence of urea or gndHCl (dotted line in **Suppl.** **Fig. 2**) is very similar to that obtained by the corresponding hydrazone formed by the reaction of RBH with diacetyl **[20]**. The maximum FSA increase of the protein carbonyl-RBH hydrazone that is caused by gndHCl may be due to its 3 amino groups compared to the 2 amino groups of the non-effective urea, and possibly because of its higher chaotropic potential than of urea **[36]**. Moreover, the FSA of the protein carbonyl-RBH hydrazone is stable at 4ºC (and at pH 5) in the dark even after 2 days (data not shown). This is expected by the known stability of the hydrazone bonds at the pH range 5 to 9 **[37]**.

The molar stoichiometry of the reaction of RBH with protein carbonyls was found to be 1:1 by the performed titration experiments (**Suppl.** **Fig. 3A,B**), and was also proven by the identical slopes of both the standard curves of RBH and of protein carbonyls (**Suppl.** **Fig. 3C**). It is also in accordance to the proposed RBH assay reaction mechanism (**Fig. 1**), which proceeds via the opening of the non-fluorescent cyclic spirolactam ring amide of RBH. This opening can take place either via a low pH-proton-induced conformation change of RBH **[20]**, or by the gndHCl in the assay buffer (33/17 mM C/P - 8 M gndHCl, pH 5) as the stoichiometry 1:1 suggests, or during the reaction of RBH (at acidic pH) with the protein carbonyls (and the formation of the hydrazone bond). Therefore, the molar carbonyl content of a protein can be easily determined fluorometrically by the RBH assay, by using an RBH standard curve the slope of which sets the sensitivity limit of the RBH assay. However, the RBH standard curve slope at 8 M gndHCl is minimum (**Suppl.** **Fig. 3C**), because its value increases ~3-fold at saturated gndHCl concentration in the C/P buffer (data not shown), which, consequently, near triples the sensitivity detection of the RBH assay (**Table 1**). At 8 M gndHCl, the detection sensitivity limit of the RBH assay can be as low as 2 nM (or 0.6 pmol in 0.3 ml, for a minimum 100 FU), and it can be even lower depending on the S/N ratio and the sensitivity of the spectrofluorometer in use. This makes it almost 600-fold more sensitive than the ntrDNPH assay **[1]**. Thus, the RBH assay represents a reliable alternative for protein samples below the ntrDNPH assay’s sensitivity limit.

In contrast, gndHCl in the FTC assay quenches the fluorescence of the protein carbonyl-FTC hydrazone **[14]**, and this may contribute to its ~11-fold lower sensitivity (carbonyl detection sensitivity limit) compared to that of the RBH assay (**Table 1**). Additionally, the combination of DOC with TCA in the precipitation of the protein sample in the RBH assay is highly efficient (~95%) even for protein samples at ~2.5 µg **[38, 39]**, which constitutes and the minimum protein quantity limit of the RBH assay. Moreover, DOC-TCA combination causes the co-precipitation and of the acid soluble proteins (they co-precipitate together with the bound on them DOC). On the other hand, TCA alone used by the FTC assay does not precipitate acidic soluble proteins, let alone the recovery of proteins by TCA precipitation alone can be as low as 24% **[18]**. Indeed, considerable protein loss (acid soluble proteins included) has been reported during precipitation with TCA alone **[17]**. Considering all these above factors and also the RBH assays’ minimum protein quantity requirement, its cumulative and functional sensitivity is 8,500- and 800-fold higher, respectively, than those of the fluorescent FTC assay (**Table 1**). The advantages of the RBH assay over the FTC assay are outlined in **Table 4**.

The RBH assay can be further simplified by omission of the DOC-TCA protein precipitation step. The unreacted RBH in the assay reaction mixture can be separated from the protein by retaining the protein fraction in the filter component of an ultrafiltration microcentrifugal filter device such as those commercially available (e.g. Amicon Ultra-0.5 ml 10K). The RBH assay may be also modified to analyze RBH-treated carbonylated proteins subjected to SDS denaturing protein gel electrophoresis. Removal of unreacted RBH from the gel can be achieved by a solvent (e.g. MetOH/acetone) wash, incubation of the gel in the 8 M gndHCl C/P buffer, pH 5, and visualization (by fluorescence emission) of the carbonyl content in protein-RBH hydrazones separated on the gel in bands. Electrophoretic separation of the proteins in the sample may be performed after or before their reaction with RBH, whereby in the latter case RBH should react with the protein bands on the gel.

***Statistical precision of the RBH assay and comparison with the ntrDNPH assay for data coincidence***: The RBH assay was also analyzed for the closeness (precision) in individual measures of protein carbonyls when applied repeatedly to multiple aliquots of a single biological sample, and between days. The *within-day* % coefficient variation for the RBH assay is <3.5% and the variance of *intermediate precision* (or *between day repeatability*) is <4.0%. The statistical coincidence of the carbonyl values obtained by the RBH and ntrDNPH assays for the same sample is tested by measuring the protein carbonyls of BSA_ox_ against BSA_red_ at various BSA_ox/red_ ratios and at a fixed total quantity (each tested in triplicate), and their means (from the triplicates) are statistically compared (similar data are obtained with lysozyme_ox/red_ and pepsin_ox/red_ protein mixtures). As shown in **Fig. 2A**, the mean values of protein carbonyls for each sample obtained by these two assays have an excellent Pearson correlation (*r* = 0.993, at confidence interval 95%). Nonetheless, this correlation actually measures the strength of the relation between the variable measured by two assays and not the agreement between them **[40]**. This is evaluated by subjecting these data to a Bland-Altman plot analysis, which shows that the differences with the RBH assay against the ntrDNPH assay are not statistically significant (**Fig. 2B**).

***Heme protein and DNA possible interference***: Heme proteins Hb and cyt.*c* were investigated for interference with the RBH assay because RBH has been used for their quantification as well. However, the presumed quantification mechanism does not involve any adduct formation between these heme proteins and RBH, but its hydrolysis to the fluorescent rhodamine B (which presumably increases its fluorescence by incorporation in the micelles of the detergent sodium dodecylbenzene sulfonate **[26, 27]**). Nonetheless, we investigated any possible interference of these heme proteins on the RBH assay as follows: We applied the RBH assay on untreated (untr) and artificially decarbonylated (NaBH_4_-reduced) Hb and cyt.*c* (Hb_untr_, cyt.*c*_untr_ and Hb_red_, cyt.*c*_red_, respectively), and compared it with the control ntrDNPH assay **[1]**. Both assays give same protein carbonyl values (in nmole mg^-1^ protein) for each of the untreated heme proteins (**Table 2**). However, only the RBH assay was able to measure the extremely low carbonyl background in Hb_red_ and cyt. *c*_red_ (64- and ~23 fold lower than that for the Hb_untr_ and cyt.*c*_untr_, respectively) due to its >800-fold more sensitive carbonyl detection limit in comparison to that of the ntrDNPH assay. For comparison, the stoichiometric 1:1 heme content of Hb and cyt.*c* (62 and 83 nmoles mg^-1^, respectively) is 1660- and 775-fold higher than the carbonyl background in Hb_red_ and cyt.*c*_red_, respectively. Nonetheless, any interference due to the absorbance of an unknown protein sample at the wavelengths used by the RBH assay can be cancelled out by the sample blank.

The RBH assay was also tested on pure DNA in order to assess any interference (**Table 2**) as result either of direct reaction (of its bases) or its non-specific association with RBH. Detailed description of the employed experiments is presented in the supplement’s *Part II. Test of DNA interference on the RBH assay***,** and explanations on the definition of this particular interference and of the results are presented in the notes of **Table 2**. Washing conditions of unreacted RBH employed in the RBH assay proved to be 100% effective; DNA interference is zero (**Table 2**; data in parentheses). This result proves that there is no direct reaction of RBH with DNA, as this was also shown with DNPH **[1]**; let alone that DNA proved to be insoluble in the protein-RBH hydrazone C/P - gndHCl solubilization buffer of the RBH assay. This finding also suggests that the only possible RBH-DNA association will be non-specific, and for the RBH assay was found to be statistically insignificant (0.03%). Therefore, no removal of DNA from protein samples is required when using the RBH assay.

***Standardization of the RBH assay on indicative samples***: The RBH assay was tested on indicative samples for carbonyl content (**Table 3**) in the cytoplasmic and histone protein fractions (isolated as described in the supplement’s *Section IV* of the ntrDNPH assay **[1]**). Histone fraction was used to also test the assay’s detection limit for protein fractions present in cells in very low quantities.

Moreover, histone fraction was also chosen in order to introduce its carbonyl concentration levels as a possible indirect indicator for the oxidative status of chromosomal (and microbial) DNA. Histones protect nuclear DNA from oxidative damage by free radicals, but they themselves are also sensitive to oxidative damage in the process. Mitochondrial DNA, on the other hand, is not protected in its close proximity to the radical production site (the electron transport chain), because of the absence of histones. Histones are the major protein components of chromatin with many roles. They are necessary for transcription and replication processes through DNA detachment/reattachment, which, however, can be influenced by the potential cross-linking of oxidatively damaged histones with DNA. Moreover, modification of histones by ROS such as H_2_O_2_ can alter their ionic charge and affect their functions in gene expression and chromatin integrity. Thus, oxidatively modified histones must be efficiently degraded by nuclear proteasomes to maintain chromatin integrity. Extensive reviews for these processes and the various roles of histones are presented elsewhere **[41-43]**. Therefore, the carbonyl content of histones can be an indicator, possibly clinical as well, of various biological processes and diseases, and its accurate determination is very important in studies that investigate the various roles of histones. Moreover, due to proximity of DNA with histones their carbonylation can be also used as an indirect indicator of DNA damage, possibly in comparison to DNA fragmentation **[44, 45]** or to other markers of DNA oxidative modification.

Histone carbonyls have been determined by the stdDNPH assay **[33]** and by immunodetection **[46]**. For example, histone carbonyls were estimated in rat nuclei by the stdDNPH assay (at ~5 and ~11 nmol mg^-1^ protein from liver and spleen, respectively), and their levels (2-fold higher than in proteins from mitochondria and cytoplasm) were attributed to an extensive modification of their lysine and arginine residues **[47]**. However, these carbonyl values were obtained by the proteins in the supernatant fraction resulting after streptomycin-DNA precipitation, and they are erroneous because they do not belong to histones since these positively charged proteins are expected to co-precipitate with DNA. The second immunodetection approach for histone carbonyl evaluation (with anti-DNP antibody on SDS-gels, and estimation by Western blotting **[46]**) is semi quantitative, and has been used to assess H1-carbonyls in bovine organs **[48]**, while a recent modification (oxy-2D-Triton-Acetic acid-Urea Western blot method) has been used to assess histone carbonylation (in arbitrary units) in proliferating cells **[49]**. Moreover, the immunodetection methods are not suitable for the accurate evaluation of the oxidative modification of histones because of other factors affecting the variability of the obtained data **[50]**. Nor are other methods that require samples with high protein content (such as the standard DNPH and FTC assays), in light of the fact that histones constitute small fraction of total proteins (especially in plant tissues).

The ultrasensitive RBH assay of the present study was shown to be the most appropriate assay for the accurate quantification of histones, especially for the small quantities that are isolated from plant tissues (**Table 3**). Indicatively, carbonylation of histones was assessed in the unstressed plants *B. oleracea* and *L. sativa*, and was 3- and 25-fold lower, respectively, than in their cytoplasmic proteins. Such difference was not observed in cytoplasmic proteins fractionated from normal rat brain stem and intestine tissue (**Table 3**). The RBH assay is also tested on carbonylated and decarbonylated control proteins such as BSA_ox/red_ (lysozyme_ox/red_, pepsin_ox/red_ were also tested), and was compared with the ntrDNPH assay on total proteins from human blood serum carbonyl content. Comparison were also made with the protein carbonyl values in human blood serum/plasma determined by the FTC assay (**Table 3**). Both RBH and ntrDNPH assays produce statistically same carbonyl values for human serum, as this was verified by the statistical comparison of both assays for data coincidence (**Fig. 2**). However, variation is observed with the carbonyl values determined by the FTC assay on human blood serum, which can be attributed to certain interfering factors stated elsewhere **[17]**.

Lastly, the RBH assay was tested on carbonylated cellulose-based polysaccharides (see supplement’s *Part III. Test of the RBH assay on carbonylated polysaccharides*), given their carbonyl content can be estimated by the control ntrDNPH assay **[1]**. It was found that the minimum required incubation time for fluorescence stabilization of the polysaccharide-RBH product was 2 hrs (in contrast to the established 1-hr incubation for protein carbonyls) to obtain a measurable FU value. However, the carbonyl content value (in nmoles carbonyls mg^-1^ polysaccharide) for CMCellu_NaClO-carbnylated_ obtained by the RBH assay was ~25 fold-lower than that obtained by the ntrDNPH assay **[1]**. Besides this inconsistency, no proportionality was obtained with the RBH assay for the various polysaccharide quantities tested, while the fluorescence of the RBH-CMCellu_NaClO-carbnylated_ product was quite unstable over time (its FU reached zero after storage for 1 day at 4°C). It is concluded that the RBH assay cannot be applied on polysaccharides, and that the ntrDNPH assay is the assay of choice.

**Tables**

| **Table 1. Sensitivity of the RBH assay vs the FTC assay** | | |
| --- | --- | --- |
| **Assays**  **Sensitivity types** | **RBH**  [FTC] | **RBH/FTC**  **sensitivity ratio** |
| A. Carbonyl detection limit (in pmoles) | 0.415^a^*  [4.4]^b^ | **10.6**  (= 4.4/0.415) |
| B. Minimum protein (in µg)^c^ | ≥2.5  [2,000] | 800  (= 2,000/2.5) |
| C. Minimum protein (in µg) per 1 nmole carbonyl at its detection limit^c^ | 6,024  (= 2.5/0.000415)  [~455,000]  (= 2,000/0.0044) | 75.5  (=455,000/6,024) |
| Cumulative sensitivity limit (= AxB) | 1.038  [8,800] | ~8,500 |
| Functional sensitivity limit (= AxC) | 2,500  2,002,000] | 800 |

^a^Values for the RBH assay are based on a sensitivity limit of 0.5 pmole RBH in 0.3 ml 33/17 mM C/P - 8 M gndHCl buffer (pH 5) that produces an accurately measured value of 100 FU by the spectrofluorometer in use (Shimadzu model RF-1501, set at 10 nm width slit and high sensitivity, using a 0.3 ml 45×4×4 mm quartz cuvette), which corresponds to an RBH standard curve equation ***Y***_FU_ = 200•***X***_pmoles_ (i.e., 1 pmole carbonyls emits 200 FU; **Suppl.** **Fig. 3C**). Given this value in a protein sample represents only 80% of the total carbonyl-RBH hydrazone carbonyls (the rest 20% is used for protein determination after reaction with RBH), the actual minimum value of sample carbonyls is 0.625 pmoles. However, the aforementioned cuvette in use can measure a minimum volume 0.2 ml (33% decrease of the 0.3 ml RBH solubilizate), and this decreases the limit of the RBH assay to 0.415 pmoles (~2 nM).

*The slope value (200) of the RBH standard curve in **Suppl.** **Fig. 3C** could become ~600 (i.e., 1 pmole carbonyls emits 600 FU; data not shown) when the 33/17 mM C/P buffer is set at gndHCl saturated concentration (data not shown). This means that the sensitivity for the RBH assay can be 3-fold higher at saturating than at 8 M gndHCl, and that the sensitivity ratios of the RBH over the FTC assay will increase accordingly.

**^b^**Numbers in brackets are for the FTC assay. Its carbonyl detection limit has been previously determined to be ~100 fold higher than the limit of the stdDNPH assay **[17]**. Since the latter limit is determined at 443 pmoles in the present study, the actual limit of the FTC assay is ~4.4 pmoles.

**^c^**Minimum sample protein quantities used in this table for RBH and FTC assays are 2.5 and 2,000 µg, respectively, with that for the FTC assay previously reported **[17]**.

| **Table 2. RBH assay interference tests** | |
| --- | --- |
| **Heme proteins** | carbonyls  nmoles mg^-1^ protein |
| Cyt.*c*_untr_^a^  Cyt.*c*_red_ | 1.15 ± 0.09 (1.09 ± 0.15)^b^  0.05 ± 0.01^c^ (0)^b^ |
| Hb_untr_^a^  Hb_red_ | 5.15 ± 0.07 (5.30 ± 0.15)^b^  0.08 ± 0.03^b^ (0)^b^ |
| **DNA: its non-specific binding with RBH^a^** | |
| Tested DNA quantity: in mg and in corresponding {nmoles carbonyls}^b^ | |
| 0.1 mg  {306}^b^ | 0.2 mg  {612}^b^ |
| (0 or 0%)^a,c^  [0.09 or 0.03%]^d^ | (0 or 0%)^a,c^  [0.18 or 0.03%]^d^ |

***Notes on heme protein interference***

^a^Carbonyl content (nmoles mg^-1^ protein) of cyt.*c*_untr_ (MW 12,000) and Hb_untr_ (MW 64,500), compared to their heme content (83 and 62 nmoles, respectively) mg^-1^, is 80- and 10-fold lower, respectively, which suggests no heme interference on the RBH assay.

^b^Values in parentheses, determined by the ntrDNPH assay, have been previously reported **[1]**.

^c^Carbonyl values for cyt.*c*_red_ and Hb_red_ obtained by the RBH assay are indicative to its very high detection limit (**Table 1**).

***Notes on DNA interference***

^a^DNA % interference is defined as the number of nmoles DNA carbonyl groups (shown by the 1^st^ number in parentheses) that are detected by the RBH assay out of theoretical 100 nmoles contained in the tested quantity of DNA. This is expressed as %, and is shown as such by the 2^nd^ number in parentheses.

^b^Values are nmoles DNA carbonyls contained in the tested DNA (0.1 and 0.2 mg), and are derived from the correspondence of 1 µg DNA to 3.06 nmoles carbonyls as shown elsewhere **[1]**.

^c^Zero values in parentheses (designating same quantities as those in parentheses explained in table’s *Note ‘a’*) are derived from the fluorescence values of the RBH assay’s solubilization solution due to the presence of solubilized DNA (after the initial solubilization of its pellet in NaOH). The obtained zero values of carbonyls are explained by the observation that the RBH assay’s pH-5-adjusted solubilization solution does not solubilize the DNA that precipitates during the application of the assay (see article’s *Part C. Standardization of the RBH assay against possible interfering factors*). Therefore, the zero value can be due either to the non-solubilization of DNA or to the overrun of the sensitivity limit of the assay to detect the RBH reagent that may be non-specifically bound on the minor quantity of DNA that may have been solubilized. DNA insolubility in the solubilization solution of the RBH assay is the main mechanism by which this assay does not exhibit RBH-DNA interference (even at its minor degree determined 0.03%) when testing carbonyls in protein samples that may be contaminated with DNA.

^d^Values in brackets (designating same quantities as those in parentheses explained in table’s *Note ‘a’*) are derived from the fluorescence values of the RBH assay’s pH-5-adjusted solubilization solution, measured after initially subjecting the insoluble DNA precipitate to NaOH solubilization (as also described in the preceding table *Note* *‘c’*), and then mixing the resulting solubilizate with the corresponding assay solubilization solutions. The DNA pellet alkaline pre-solubilization procedure is employed in order to determine the degree (0.03%) of the non-specific interference of DNA on the RBH assay.

| **Table 3. Standardization of the RBH assay by control proteins and indicative samples, and vs the ntrDNPH assay** | | | | |
| --- | --- | --- | --- | --- |
| **Assay**  **Samples** | | **RBH** | | |
|  |  | nmoles mg^-1^ protein | | |
| **Control proteins** | | | | |
| BSA_ox_^a^  BSA_red_^a^ | | 22.9 ± 1.8  ~0 | | |
| **Indicative sample proteins^b^: cytoplasmic / histone (ratio)** | | | | |
| Cauliflower *B. oleracea* (C*Bo*)  C*Bo*_untr_  C*Bo*_red_ | | 9.3 ± 0.79 / 1.7 ± 0.11 (5.5)  ~0 | | |
| Lettuce *L. sativa* (L*Ls*)  L*Ls*_untr_  L*Ls*_red_ | | 24.9 ± 1.9 / 1.0 ± 0.09 (25)  ~0 | | |
| Rat Brain Stem (RBS)  RBS_untr_  RBS_red_ | | 0.62 ± 0.05 / 0.60 ± 0.07 (1)  ~0 | | |
| Rat Intestine (RI)  RI_untr_  RI_red_ | | 0.71 ± 0.09 / 0.56 ± 0.06 (1.3)  ~0 | | |
| **Human blood protein carbonyls** | | | | |
| Serum carbonyls^c^ | | | | **FTC assay**^f^ |
| **Assays**  **Subjects**^c^ | **RBH** | | **ntrDNPH**^c^ |  |
| Males  Females | 1.08 ± 0.07^c,d,e^  1.14 ± 0.09^c,d,e^ | | 1.09 ± 0.07^c,d,e^  1.17 ± 0.09^c,d,e^ | ~0.43 **[17]** |

^a^Control lysozyme_ox/red_, pepsin_ox/red_ were also tested and gave analogous results (data not shown).

^b^Values for cytoplasmic and histone protein fractions are shown by the numbers left and right to the separating slash, respectively, while the values in parenthesis represent their cytoplasmic/histone carbonyl content ratio. Histone carbonyls were not detected by the much lower in sensitivity ntrDNPH assay **[1]**. Values are averages from at least 5 independent measurements (SD, not shown, is < 10% of the average).

^c^Values (in nmoles mg^-1^) are averages (± SD) from 10 male and 10 middle age female middle age subjects. Values by the ntrDNPH assay are from previous study **[1]**.

^d^Comparison of the new assays for the same and between sex; there is no statistical difference for values statistically significant at p<0.05.

^e^Comparison between males and females for the three assays; there is a ~30% statistical difference for the stdDNPH assay for values statistically significant at p<0.05.

^f^Carbonyl values determined by the FTC assay (on plasma from unspecified gender) are listed for comparison with the RBH assay.

*IMPORTANT NOTE: The present study did not perform the FTC assay because the assumed by the assay stoichiometry 1:1 for the protein carbonyl-FTC hydrazone and the FTC reagent (used to construct the assay’s standard curve* ***[17]****) is not supported by the referred literature* ***[14]****.*

| **Table 4. Advantages of the RBH assay over the FTC assay** | |
| --- | --- |
| **RBH assay** | **FTC assay** |
| The fluorescence of the protein carbonyl-RBH hydrazone is greatly increased by gndHCl | The fluorescence of the protein carbonyl-FTC hydrazone is quenched by gndHCl |
| Requires samples with minute protein quantity (≥2.5 µg), and is only limited by the protein recovery of its DOC-TCA precipitation step (see below) and by the sensitivity limit of the assay. The assay’s minimum protein limit of 2.5 µg is possible because of the use of an ultrasensitive protein quantification assay **[32]**. The RBH assay can use protein quantities even lower than 2.5 µg if its DOC-TCA protein precipitation step is replaced by the use of microcentrifugal filter devices (e.g. Amicon Ultra-0.5 ml 10K), described in **step 1.1** of the article’s *Part A. Protocol of the RBH assay* | Requires samples of high protein concentration (2-10 mg ml^-1^). Accounting the fluorescence quenching by gndHCl of the carbonyl-FTC hydrazone and the at least 2,000-fold higher protein quantity limit of the FTC assay, its cumulative and functional sensitivity is at least 8,500- and 800-fold lower, respectively than those of the RBH assay (**Table 1**) |
| Protein recovery of the RBH assay’s DOC-TCA precipitation step is ≥90% even for protein samples as low as 2 to 5 µg **[38, 39]**. Since DOC co-precipitates also acid soluble proteins, the carbonyls of such proteins are also measured by the RBH assay | Given that the recovery of proteins by TCA precipitation can be as low as 24% **[18]**, protein recovery by the FTC assay’s TCA precipitation step is not only very ineffective but the acid soluble proteins in the sample are also lost. All these add up to assay’s unreliability and very low sensitivity |
| Very short assay reaction incubation time (1 hr); 24-fold shorter than that of the FTC assay | Very long assay reaction incubation time (overnight, up to 24 hrs) |
| Stable fluorescent hydrazone (for 2 days in the dark at RT). | Unstable fluorescent hydrazone |
| High reproducibility, no DNA interference | Low reproducibility, DNA interference |

**Caveats**

Interfering reagents possibly present in various sample treatments can be canceled out by sample dilution and/or by testing in the appropriate sample blank. For example, DTT (used to pretreat the protein sample by a standardized procedure mentioned in the article’s *Part A. Protocol of the RBH assay*) does not interfere at 25 mM in the RBH assay reaction mixture.

**Acknowledgments**

This work was financially supported by the Greek Ministry of Education.

**Declarations of interest**: none

**References**

[1] Georgiou, C. D.; Zisimopoulos, D.; Argyropoulou, V.; Kalaitzopoulou, E.; Salachas, G.; Grune, T. Protein and cell wall polysaccharide carbonyl determination by a neutral pH 2,4-dinitrophenylhydrazine-based photometric assay. *Redox Biology,* DOI: https://doi.org/10.1016/j.redox.2018.04.010 2018.

[2] Daneshvar, B.; Frandsen, H.; Autrup, H.; Dragsted, L. O. gamma-Glutamyl semialdehyde and 2-amino-adipic semialdehyde: biomarkers of oxidative damage to proteins. *Biomarkers* **2:** 117-123; 1997.

[3] Purdel, N. C.; Margina, D.; Ilie, M. Current methods used in the protein carbonyl assay. *Annual Research & Review in Biology* **4:** 2015-2026; 2014.

[4] Sorolla, M. A.; Rodríguez-Colman, M. J.; Tamarit, J.; Ortega, Z.; Lucas, S. J. J.; Ferrer, I.; Ros, J.; Cabiscol, E. Protein oxidation in Huntington disease affects energy production and vitamin B6 metabolism. *Free Radic. Biol. Med.* **49:** 612-621; 2010.

[5] Irazusta, V.; Obis, E.; Moreno-Cermeño, A.; Cabiscol, E.; Ros, J.; Tamarit , J. Yeast frataxin mutants display decreased superoxide dismutase activity crucial to promote protein oxidative damage. *Free Radic. Biol. Med.* **48:** 411-420.; 2010.

[6] Roe, M. R.; Xie, H.; Bandhakavi, S.; Griffin, T. J. Proteomic mapping of 4-hydroxynonenal protein modification sites by solid-phase hydrazide chemistry and mass spectrometry. *Anal. Chem.* **79:** 3747-3756; 2007.

[7] Hensley, K. Detection of protein carbonyls by means of biotin hydrazide-streptavidin affinity methods. *Methods Mol. Biol.* **536:** 457-462; 2009.

[8] Vemula, V.; Ni, Z.; Fedorova, M. Fluorescence labeling of carbonylated lipids and proteins in cells using coumarin-hydrazide. *Redox Biol.* **5:** 195-204; 2015.

[9] Zhu, X.; Shinohara, H. Fluorescence enhancement of fluorescent unnatural streptavidin by binding of a biotin analogue with spacer tail and its application to biotin sensing. *The Scientific World Journal* **2014:** ID 165369, 6 p; 2014.

[10] Caperna, T. J.; Shannon, A. E.; Blomberg, L. A.; Garrett, W. M.; Ramsay, T. G. Identification of protein carbonyls in serum of the fetal and neonatal pig. *Comp. Biochem. Physiol. B Biochem. Mol. Biol.* **156:** 189-196; 2010.

[11] Vásquez-Garzón, V. R.; Rouimi, P.; Jouanin, I.; Waeg, G.; Zarkovic, N.; Villa-Treviño, S.; Guéraud, F. Evaluation of three simple direct or indirect carbonyl detection methods for characterization of oxidative modifications of proteins. *Toxicol. Mech. Methods.* **22:** 296-304; 2012.

[12] Shacter, E. Quantification and significance of protein oxidation in biological samples. *Drug Metab. Rev.* **32:** 307-326; 2000.

[13] Tamarit, J.; de Hoogh, A.; Obis, E.; Alsina, D.; Cabiscol, E.; Ros, J. Analysis of oxidative stress-induced protein carbonylation using fluorescent hydrazides. *J. Proteomics* **75:** 3778-3788; 2012.

[14] Ahn, B.; Rhee, G. S.; Stadtman, R. E. Use of fluorescein hydrazide and fluorescein thiosemicarbazide reagents for the fluorometric determination of protein carbonyl groups and for the detection of oxidized protein on polyacrylamide gels. *Anal. Biochem.* **161:** 245-257; 1987.

[15] Mohanty, J. G.; Jaffe, J. S.; Schulman, E. S.; Raible, D. G. A highly sensitive fluorescent micro-assay of H_2_O_2_ release from activated human leukocytes using a dihydroxyphenoxazine derivative. *J. Immunol. Meth.* **202:** 133-141; 1997.

[16] Chaudhuri, A. R.; de Waal, E. M.; Pierce, A.; Van Remmen, H.; Ward, W. F.; Richardson, A. Detection of protein carbonyls in aging liver tissue: A fluorescence-based proteomic approach. *Mech. Ageing Dev.* **127:** 849-861; 2006.

[17] Mohanty, J. G.; Bhamidipaty, S.; Evans, M. K.; Rifkind, J. M. A fluorimetric semi-microplate format assay of protein carbonyls in blood plasma. *Anal. Biochem.* **400:** 289-294; 2010.

[18] Fic, E.; Kedracka-Krok, S.; Jankowska, U.; Pirog, A.; Dziedzicka-Wasylewska, M. Comparison of protein precipitation methods for various rat brain structures prior to proteomic analysis. *Electrophoresis* **31:** 3573-3579; 2010.

[19] Eldridge, G. M.; Weiss, G. A. Hydrazide reactive peptide tags for site-specific protein labeling. *Bioconjug. Chem.* **22:** 2143-2153; 2011.

[20] Li, X.; Duerkop, A.; Wolfbeis, O. S. A fluorescent probe for diacetyl detection. *J. Fluoresc.* **19:** 601-606; 2009.

[21] Li, P.; Ding, G.; Deng, Y.; Punyapitak, D.; Li, D.; Cao, Y. Determination of malondialdehyde in biological fluids by high-performance liquid chromatography using rhodamine B hydrazide as the derivatization reagent. *Fee Radic. Biol. Med.* **65:** 224-231; 2013.

[22] Yang, X. F.; Guo, X. Q.; Zhao, Y. B. Development of a novel rhodamine-type fluorescent probe to determine peroxynitrite. *Talanta* **57:** 883-890; 2002.

[23] Chen, X.; Zou, J. Application of rhodamine B hydrazide as a new fluorogenic indicator in the highly sensitive determination of hydrogen peroxide and glucose based on the catalytic effect of iron(III)-tetrasulfonato-phthalocyanine. *Microchim. Acta* **157:** 133-138; 2007.

[24] Dujols, V.; Ford, F.; Czarnik, A. W. A long-wavelength fluorescent chemodosimeter selective for Cu(II) ion in water. *J. Am. Chem. Soc.* **119:** 7386-7387; 1997.

[25] Wu, C. M.; Chen, Y. H.; Dayananda, K.; Shiue, T. W.; Hung, C. H.; Liaw, W. F.; Chen, P. Y.; Wang, Y. M. Sensitivity evaluation of rhodamine B hydrazide towards nitric oxide and its application for macrophage cells imaging. *Anal. Chim. Acta.* **708(1-2):** 141-148; 2011.

[26] Yang, X.-F.; Guo, X.-Q.; Li, H. Fluorimetric determination of hemoglobin using spiro form rhodamine B hydrazide in a micellar medium. *Talanta* **61:** 439-445; 2003.

[27] Shen, Y.; Yang, X. F.; Wu, Y.; Li, C. Sensitive spectrofluorimetric determination of cytochrome C with spirocyclic rhodamine B hydrazide in micellar medium. *J. Fluoresc.* **18:** 163-168; 2008.

[28] Alvarez, B.; Radi, R. Peroxynitrite reactivity with amino acids and proteins. *Amino Acids* **25(3-4):** 295-311; 2003.

[29] Grintzalis, K.; Zisimopoulos, D.; Grune, T.; Weber, D.; Georgiou, C. D. Method for the simultaneous determination of free/protein malondialdehyde and lipid/protein hydroperoxides. *Free Radic. Biol. Med.* **59:** 27-35; 2013.

[30] Nelson, K. J.; Parsonage, D. Measurement of peroxiredoxin activity. *Curr. Protoc. Toxicol.* **49:7.10:** 7.10.1–7.10.28; 2011.

[31] Florence, T. M. Degradation of protein disulphide bonds in dilute alkali. *Biochem. J.* **189:** 507-520; 1980.

[32] Georgiou, C. D.; Grintzalis, K.; Zervoudakis, G.; Papapostolou, I. Mechanism of Coomassie brilliant blue G-250 binding to proteins: a hydrophobic assay for nanogram quantities of proteins. *Anal. Bioanal. Chem.* **391:** 391-403; 2008.

[33] Levine, R. L.; Garland, D.; Oliver, N. C.; Amici, A.; Climent, I.; Lenz, G.-A.; Ahn, W.-B.; Shaltiel, S.; Stadtman, R. E. Determination of carbonyl content in oxidatively modified proteins. *Methods Enzymol.* **186:** 464-479; 1990.

[34] Cao, G.; Cutler, R. G. Protein oxidation and aging. I. Difficulties in measuring reactive protein carbonyls in tissues using 2,4-dinitrophenylhydrazine. *Arch. Biochem. Biophys.* **320:** 106-114; 1995.

[35] Luo, S.; Wehr, N. B. Protein carbonylation: avoiding pitfalls in the 2,4-dinitrophenylhydrazine assay. *Redox Rep.* **14:** 159-166; 2009.

[36] Monera, O. D.; Kay, C. M.; Hodges, R. S. Protein denaturation with guanidine hydrochloride or urea provides a different estimate of stability depending on the contributions of electrostatic interactions. *Protein Sci.* **3:** 1984-1991; 1994.

[37] Kalia, J.; Raines, R. T. Hydrolytic stability of hydrazones and oximes. *Angew Chem. Int. Ed. Engl.* **47:** 7523-7526; 2008.

[38] Arnold, U.; Ulbrich-Hofmann, R. Quantitative protein precipitation from guanidine hydrochloride-containing solutions by sodium deoxycholate/trichloroacetic acid. *Anal. Biochem.* **271:** 197-199; 1999.

[39] Bensadoun, A.; Weinstein, D. Assay of proteins in the presence of interfering materials. *Anal. Biochem.* **70:** 241-250; 1976.

[40] Bland, J. M.; Altman, D. G. Statistical methods for assessing agreement between two methods of clinical measurement. *Lancet* **1:** 307-310; 1986.

[41] Jung, T.; Catalgol, B.; Grune, T. The proteasomal system. *Mol. Asp. Med.* **30:** 191-296; 2009.

[42] Jung, T.; Höhn, A.; Grune, T. The proteasome and the degradation of oxidized proteins: Part III—Redox regulation of the proteasomal system. *Redox Biol.* **2:** 388-394; 2014.

[43] Chondrogianni, N.; Petropoulos, I.; Grimm, S.; Georgila, K.; Catalgol, B.; Friguet, B.; Grune, T.; Gonos, E. S. Protein damage, repair and proteolysis. *Mol. Asp. Med.* **35:** 1-71; 2014.

[44] Georgiou, D. C.; Patsoukis, N.; Papapostolou, I. Assay for the quantification of small-sized fragmented genomic DNA. *Anal. Biochem.* **339:** 223-230; 2005.

[45] Georgiou, D. C.; Papapostolou, I.; Grintzalis, K. Protocol for the quantitative assessment of DNA concentration and damage (fragmentation and nicks). *Nature Protocols* **4:** 125-131; 2009.

[46] Shacter, E.; Williams, J. A.; Lim, M.; Levine, R. L. Differential susceptibility of plasma proteins to oxidative modification: examination by western blot immunoassay. *Free Radic. Biol. Med.* **17:** 429-437; 1994.

[47] Pleshakova, O. V.; Kutsyi, M. P.; Sukharev, S. A.; Sadovnikov, V. B.; Gaziev, A. I. Study of protein carbonyls in subcellular fractions isolated from liver and spleen of old and *γ*-irradiated rats. *Mech. Ageing Dev.* **103:** 45-55; 1998.

[48] Wondrak, G. T.; Cervantes-Laurean, D.; Jacobson, E. L.; Jacobson, M. K. Histone carbonylation *in vivo* and *in vitro*. *Biochem. J.* **351:** 769-777; 2000.

[49] García-Giménez, J. L.; Ledesma, A. M.; Esmoris, I.; Romá-Mateo, C.; Sanz, P.; Viña, J.; Pallardó, F. V. Histone carbonylation occurs in proliferating cells. *Free Radic. Biol. Med.* **52:** 1453-1464; 2012.

[50] Augustyniak, E.; Adam, A.; Wojdyla, K.; Rogowska-Wrzesinska, A.; Willetts, R.; Korkmaz, A.; Atalay, M.; Weber, D.; Grune, T.; Borsa, C.; Gradinaru, D.; Chand Bollineni, R.; Fedorova, M.; Griffiths, H. R. Validation of protein carbonyl measurement: a multi-centre study. *Redox Biol.* **4:** 149-157; 2015.

**Figure legends**

**Fig. 1**. RBH assay mechanism. Formation of a fluorescent protein carbonyl-RBH hydrazone by the reaction of protein carbonyls with RBH, and subsequent opening of the RBH spirocyclic lactam ring. The non-fluorescent spirolactam amide ring of free RBH is also opened by the assay buffer 33/17 mM C/P - 8 M gndHCl, pH 5, resulting in a fluorescent RBH hydrazine at a 1:1 molar fluorescence equivalence with the protein carbonyl-RBH hydrazone.

**Fig. 2.** Statistical data coincidence comparisons between the RBH and the ntrDNPH assay. A) Plot of BSA_ox/red_ (i.e. BSA_ox_ in various mixture ratios with BSA_red_) carbonyl values obtained by the RBH assay (solid squares) and the ntrDNPH assay (open squares). The Pearson correlation (*r*) is 0.993 (at confidence interval 95%), and the solid line represents the line of identity. B) The Bland-Altman plot, with the dotted lines indicating 95% limits of agreement (-0.3927 to 0.6335) and the thin solid line the mean difference (bias = 0.1182, with SD of bias 0.2629). Similar data (not shown) are obtained with lysozyme_ox/red_ and pepsin_ox/red_ protein mixtures.

**SUPPLEMENT**

**Protein carbonyl determination by a rhodamine B hydrazide-based fluorometric assay**

**Christos D. Georgiou, Dimitrios Zisimopoulos, Vasiliki Argyropoulou, Electra Kalaitzopoulou, Panayiotis V. Ioannou, George Salachas, Tilman Grune**

**Corresponding author:** Christos D. Georgiou

**Part I. Standardization of the RBH assay**

The following parameters of the RBH assay were investigated:

**Section I. Preparation of control protein solutions**

The RBH assay is developed on stock solutions of artificially carbonylated (oxidized; ox) control proteins (e.g., BSA_ox_ at 32 mg ml^-1^) in comparison to their non-carbonylated state (by elimination of any background carbonyl groups via NaBH_4_ reduction (BSA_red_ at 80 mg ml^-1^). Control Stock solution preparation and carbonyl content determination (of e.g. BSA_ox_ stock at 23 nmoles mg^-1^) are determined by the ntrDNPH assay previously reported **[1]**. Control lysozyme_ox/red_, pepsin_ox/red_ were also tested. The RBH assay was also standardized on indicative protein samples (from cauliflower, lettuce, and rat brain stem and intestine, untreated and NaBH_4_-decarboxylated; see data in **Table 3**). Possible heme protein interference on the RBH assay was tested with cyt.*c* and Hb (both at 2 mg ml^-1^), untreated (cyt.*c*_untr_, Hb_untr_) and NaBH_4_-reduced (cyt.*c*_red_, Hb_red_, following for their preparation the same procedure for BSA_red_); see data in **Table 2.**

**Section II. Effect of gndHCl and urea on hydrazone formation by RBH vs assay pH, reaction time, and stability of the protein carbonyl-RBH hydrazone**

Optimum assay conditions are determined for the minimum time of the formation of the protein carbonyl-RBH hydrazone resulting from the reaction of BSA_ox_ (20 µg) with RBH (at 25 µM) in comparison to RBH’s reaction conditions initially established for diacetyl **[2]**. These include incubation of control protein carbonyls in 33/17 mM citrate/phosphate (C/P) buffer, pH 3, for up to 3 hr at 37^o^C; assay pH 3 was selected (among a 2 to 5 pH range) as optimum for minimizing RBH hydrolysis (i.e. the ring opening of RBH’s spirolactam amide which results in a fluorescent product **[2]**), thereby preserving it to react with the protein carbonyls. These conditions were modified, in the present study, by testing in the C/P buffer various concentrations of gndHCl or urea (against their absence as control; **Suppl. Fig. 1**). Subsequently, the protein-RBH reaction procedure is extended to the separation of free RBH from the protein carbonyl-RBH hydrazone by its precipitation via a combination of DOC with TCA, subsequent cold acetone-wash of the resulting protein pellet (to remove any non-specifically bound free RBH), its solubilization in 33/17 mM C/P - 8 M gndHCl buffer, pH 5 (its constitution is determined in the following *Section III*), and measure of the fluorescence units (FU) of the protein carbonyl-RBH hydrazone, and its (fluorescence) stability over time.

***Reagent setup***

- **66/34 mM Citrate/Phosphate (C/P) buffer, pH 3:** Dissolve 0.059 g K_2_HPO_4_ and 0.139 g citric acid monohydrate in ddH_2_O to final 10 ml, and adjust to pH 3 (with 10 M KOH dropwise).
- **66/34 mM C/P - gndHCl buffers, pH 3:** For each 10 ml C/P - gndHCl buffer made with 0.059 g K_2_HPO_4_ and 0.139 g citric acid monohydrate, dissolve also 0.192, 1.92 or 5.72 g gndHCl (for 0.2, 2 or 6 M, respectively) in ddH_2_O to final 10 ml, and adjust to pH 3 (dropwise with 10 M KOH or HCl).
- **66/34 mM C/P - Urea buffers, pH 3:** For each 10 ml C/P - urea buffer made with 0.059 g K_2_HPO_4_ and 0.139 g citric acid monohydrate, dissolve also 0.12, 1.2 or 3.6 g urea (for 0.2, 2 or 6 M, respectively) in ddH_2_O to final 10 ml, and adjust to pH 3 (dropwise with 10 M KOH or HCl).
- **33/17 mM C/P - 8 M gndHCl buffer, pH 5:** Dissolve 29.5 mg K_2_HPO_4_, 69.5 mg citric acid monohydrate, and 7.64 g gndHCl (for 8 M) in ddH_2_O to final 10 ml, and adjust to pH 5.
- **1 mM RBH**: Dissolve 1 mg RBH in 2 ml absolute EtOH. The RBH stock solution should be always kept light-protected (e.g. wrapped in aluminum foil) and placed in an ice-water bath during use both in the RBH assay and for preparing the RBH standard curve (in *Section IV*). It can be stored at -20^o^C for at least a month.
- **210 µg ml^-1^ BSA_ox_**: Mix 10 μl 32 mg ml^-1^ BSA_ox_ stock with 1.51 ml ddH_2_O.
- **210 µg ml^-1^ BSA_red_:** Dilute from the 10-mg ml^-1^ BSA_red_ stock with ddH_2_O.
- **100% TCA:** Dissolve 10 g TCA in ddH_2_O to final 10 ml.
- **1% DOC**: Dissolve 0.01 g DOC in ddH_2_O to final 1 ml.
- **100% Acetone**: Keep cold at -20°C before use.

***Procedure***: For each 33/17 mM C/P (±gndHCl or ±urea) buffers, pH 3, mix 100 μl of their 66/34 mM stock buffers, pH 3, with 96 μl 210 µg ml^-1^ BSA_ox_ (or BSA_red_) and 5 μl 1 mM RBH (using as sample blank absolute EtOH in place of RBH), and incubate for 3 hrs at 37^o^C. To precipitate the formed BSA_ox_ carbonyl-RBH hydrazone, a modification was used of the TCA in the presence of DOC protein precipitation method (precipitates as low as 2.5 µg protein by a ≥98% efficiency **[3, 4]**) as follows: To the 196 μl mixture 4 μl 1% DOC (final 0.02%) is added, and incubated for 10 min at RT, after which 22 μl 100% TCA (final 10%) is added, and incubated for 15 min in an ice-water bath, followed by precipitation of the BSA_ox_ hydrazone with centrifugation at 16,000 *g* for 5 min at 4ºC. Then, the supernatant is discarded carefully as not to disturb the small protein pellet, which is subsequently washed 2x with 0.5 ml cold acetone (by vortexing), each time followed by centrifugation at 16,000 *g* for 5 min at 4ºC, and air-drying of the washed pellet for 10 min at RT (or drying in a stream of nitrogen) to remove any remnant by e. Finally, the resulting protein pellets for samples, S, and sample blanks, SB, are solubilized in 0.3 ml 33/17 mM C/P - 8 M gndHCl buffer, pH 5 (determined in the following *Section III*), and their fluorescence units (FU) are measured at ex/em 560/585 nm (in the spectrofluorometer Shimadzu, model RF-1501, set at 10 nm width slit and high sensitivity) against the 33/17 mM C/P - 8 M gndHCl buffer, pH 5. BSA_ox_ carbonyl-RBH hydrazone net FU (= FU_S_ - FU_SB_) are expressed as fluorescence specific activity (FSA; defined as FU µg^-1^ BSA_ox_), after quantification of the protein in each hydrazone solubilization buffer by the CBB G-250-based protein assay **[5]**. Having chosen 0.1 M gndHCl (**Suppl. Fig. 1**) in the reaction mixture of the RBH assay, BSA_ox_ reaction with RBH is tested at various incubation times (30-180 min) in order to establish the minimum assay reaction time for maximum hydrazone formation (i.e., maximum FSA value), Additionally, the stability of the protein hydrazone (i.e the stability of its FSA value) is tested up to 2 days at 4ºC in the dark. Similar data (not shown) were also obtained with pepsin_ox_ and lysozyme_ox_.

**Section III. Protein carbonyls-RBH hydrazone fluorescence dependence on pH, gndHCl and urea**

Maximization of the FSA of the protein carbonyl-RBH hydrazone is tested as a function of pH and in the presence of various gndHCl or urea concentrations (using their absence as control) in the C/P solubilization buffer of the (DOC-TCA-pelleted) protein carbonyl-RBH hydrazone **(Suppl. Fig. 2**). Protein hydrazone is formed by reaction of 20 µg BSA_ox_ (pepsin_ox_ and lysozyme_ox_ were also tested) with 25 µM RBH in the assay reaction buffer 33/17 mM C/P, pH 3**,** at 37^o^C for 1 hr (established in *Section II*) using 3 hrs incubation as additional control.

***Reagent setup***

- **66/34 mM C/P buffer, pH 3:** 10 ml, as in *Section II*.
- **33/17 mM C/P buffer, pH 2, 3, 5, 7:** Dissolve 0.295 g K_2_HPO_4_ and 0.695 g citric acid monohydrate in ddH_2_O (~90 ml) to final 100 ml, and adjust to the indicated pH’s.
- **33/17 mM C/P - GndHCl buffer, pH 2, 3, 5, 7:** For each 10 ml C/P-pH (2, 3, 5, 7) buffer made with 29.5 mg K_2_HPO_4_ and 69.5 mg citric acid monohydrate, dissolve also 2.86, 4.77, 5.73 or 7.64 g gndHCl (for 3, 5, 6 or 8 M, respectively) in ddH_2_O to final 10 ml, and adjust to the indicated pH’s.
- **33/17 mM C/P - Urea buffer, pH 2, 3, 5, 7:** For each 10 ml C/P-pH (2, 3, 5, 7) buffer made with 29.5 mg K_2_HPO_4_ and 69.5 mg citric acid monohydrate, dissolve also 1.8, 3, 3.6 or 4.8 g urea (for 3, 5, 6 or 8 M, respectively) in ddH_2_O to final 10 ml, and adjust to the indicated pH’s.
- **1 mM RBH**: As in *Section II*.
- **210 µg ml^-1^ BSA_ox_**: As in *Section II*.
- **100% TCA:** As in *Section II*.
- **1% DOC**: As in *Section II*.
- **100% Acetone**: As in *Section II*.

***Procedure***: For each 33/17 mM C/P ±gndHCl or ±urea buffer, pH 2, 3, 5 and 7, mix 100 μl 66/34 mM C/P buffer, pH 3, with 96 μl 210 µg ml^-1^ BSA_ox_ and 5 μl 1 mM RBH (i.e. assay reaction volume = 0.2 ml) and incubate for 1 and 3 hr at 37^o^C. As sample blank (SB) use absolute EtOH in place of RBH. To precipitate the so formed hydrazone by DOC-TCA in the assay reaction mixture (196 μl), 4 μl 1% DOC (final 0.02%) is added, and incubated for 10 min at RT, after which 22 μl 100% TCA (final 10%) is added, and incubated for 15 min in an ice-water bath, followed by precipitation of the BSA_ox_ hydrazone with centrifugation at 16,000 *g* for 5 min at 4ºC. Consequently, the supernatant is discarded carefully as not to disturb the small protein pellet, which is subsequently washed 2x with 0.5 ml cold acetone (by vortexing), each time followed by centrifugation at 16,000 *g* for 5 min at 4ºC (to remove any unreacted RBH), and air-drying of the washed pellet for 10 min at RT (to remove any remnant acetone; the pellet can be also dried in a stream of nitrogen). Finally, the resulting pellets for samples, S, and sample blanks, SB, are solubilized in 0.3 ml of their pH corresponding 33/17 mM C/P (±gndHCl or ±urea) buffer (pH 2, 3, 5 and 7), and the net BSA_ox_ carbonyl-RBH hydrazone FU is expressed as FSA (FU µg^-1^ BSA_ox_) after measuring their FU_S_ and FU_SB_ (as in *Section II*). Similar data (not shown) were also obtained with pepsin_ox_ and lysozyme_ox_.

**Section IV. Molar stoichiometry of the reaction between RBH and protein carbonyl groups: RBH assay standard curve**

The molar stoichiometry for the reaction of RBH and protein carbonyls is determined at 25 µM RBH in 33/17 mM C/P - 0.1 M gndHCl buffer (pH 3) for 1 hr incubation at 37^o^C, using protein hydrazone solubilization buffer 33/17 mM C/P - 8 M gndHCl, pH 5 (these assay conditions are already established in *Sections II*, *III*). The molar stoichiometry is determined by the following two independent titration schemes: Titration (A) of a constant amount (0.6 nmoles) RBH with various nmoles BSA_ox_ carbonyls, and Titration (B) of a constant amount (0.6 nmoles) BSA_ox_ carbonyls with various nmoles of RBH. In addition, the molar fluorescence of the protein carbonyl-RBH hydrazone is correlated with that of free RBH (by a standard curve of RBH solubilized under the exact protein carbony-RBH hydrazone solubilization conditions established in *Section III*) in order to be used for the determination of the carbonyl content of proteins (**Suppl. Fig. 3**). Similar data (not shown) were also obtained with pepsin_ox_ and lysozyme_ox_.

***Titration A*:**

***Reagent setup***

- **66/34 mM C/P - 0.2 M gndHCl buffer, pH 3:** Dissolve 0.059 g K_2_HPO_4_, 0.139 g citric acid monohydrate, and 0.192 g gndHCl in ddH_2_O to final 10 ml, and adjust to pH 3 (dropwise with 10 M KOH or HCl).
- **1 mM RBH**: As in *Section II*.
- **1.84, 0.9, 0.45, 0.362, 0.317, 0.27, 0.226, 0.18, 0.09 and 0.052 mg ml^-1^** **BSA_ox_ solutions:** By diluting the 32-mg ml^-1^ BSA_ox_ stock (contains 23 nmoles carbonyl mg^-1^; *Section I*).
- **100% TCA:** As in *Section II*.
- **1% DOC**: As in *Section II*.
- **100% Acetone**: As in *Section II*.
- **33/17 mM C/P - 8 M gndHCl buffer, pH 5**: As in *Section III*.

***Procedure***: In a series of 1.5-ml microcentrifuge tubes 100 μl 66/34 mM C/P - 0.2 M gndHCl buffer, pH 3, are mixed with 96 μl of the corresponding 1.84-0.052 mg ml^-1^ BSA_ox_ solutions, then to each tube 5 μl 1 mM RBH is added and incubated for at least 3 hours (long incubation time is used to allow for the complete reaction of the low (<25 µM) concentrations of RBH used in the assay reaction mixtures). For precipitation of the resulting protein-RBH hydrazone by DOC-TCA, to each assay reaction mixture 4 μl 1% DOC is added and incubated for 10 min at RT, and then 22 μl 100% TCA is added and incubated for 15 min in an ice-water bath, followed by centrifugation at 16,000 *g* for 5 min at 4ºC. Then, the supernatant is carefully discarded, and the protein pellet is washed 2x with 0.5 ml cold acetone by vortexing and centrifugation at 16,000 *g* for 5 min at 4ºC, followed by air-drying of the pellet remnant acetone for 10 min at RT (or by drying in a stream of nitrogen). The resulting sample (S) hydrazone and the corresponding sample blank (SB; containing absolute EtOH in place of RBH) pellet are solubilized each in 0.3 ml 33/17 mM C/P - 8 M gndHCl buffer, pH 5, and their FU are measured at ex/em 560/585 nm (as in *Section III*) against 33/17 mM C/P - 8 M gndHCl buffer, pH 5. The net BSA_ox_ carbonyl-RBH hydrazone fluorescence (= FU_S_ - FU_SB_) is expressed in terms of FSA (**Suppl. Fig. 3A**) as in *Section II*.

***Titration B*:**

***Reagent setup***

- **66/34 mM C/P- 0.2 M gndHCl buffer, pH 3:** As in Titration A, *Reagent setup*.
- **1 mM RBH**: As in *Section II*.
- **0.5, 0.25, 0.2, 0.175, 0.15, 0.125, 0.1 and 0.05 mM RBH solutions**: Prepare by dilution (with ddH_2_O) of the 1 mM RBH stock.
- **270 µg ml^-1^ BSA_ox_**: By dilution of the 32 mg ml^-1^ BSA_ox_ stock with ddH_2_O.
- **100% TCA:** As in *Section II*.
- **1% DOC**: As in *Section II*.
- **100% Acetone**: As in *Section II*.
- **33/17 mM C/P - 8 M gndHCl buffer, pH 5**: As in *Section III*.

***Procedure***: In a series of 1.5-ml microcentrifuge tubes 100 μl 66/34 mM C/P - 0.2 M gndHCl buffer, pH 3, is mixed with 96 μl 270 µg ml^-1^ BSA_ox_ solutions, then to each tube 5 μl of the corresponding 0.05-0.5 mM RBH solutions is added and incubated for at least 3 hours. To precipitate the resulting protein carbonyl-RBH hydrazone by DOC-TCA, to each assay reaction mixture 4 μl 1% DOC is added and incubated for 10 min at RT, and then 22 μl 100% TCA is added and incubated for 15 min in an ice-water bath, followed by centrifugation at 16,000 *g* for 5 min at 4ºC. Then, the supernatant is discarded carefully, and the protein pellet is washed 2x with 0.5 ml cold acetone by vortexing and centrifugation at 16,000 *g* for 5 min at 4ºC, followed by air-drying of the pellet remnant acetone for 10 min at RT. The resulting sample (S) hydrazone and the corresponding common sample blank (SB; containing absolute EtOH in place of RBH) pellet is solubilized in 0.3 ml 33/17 mM C/P - 8 M gndHCl buffer, pH 5, and its FU is measured at ex/em 560/585 nm (as in Titration A *Procedure*) against 33/17 mM C/P - 8 M gndHCl buffer, pH 5. The net BSA_ox_ carbonyl-RBH hydrazone fluorescence (= FU_S_ - FU_SB_) is expressed in terms of FSA (**Suppl. Fig. 3B**) as in *Section II*.

***RBH standard curve***: A concentration series (0-100 nM) of RBH (MW = 456.58) solutions is prepared by serial dilutions of of 0.1 mM RBH (starting solution, made by 10-fold dilution of the 1 mM RBH stock solution with ddH_2_O), using the 33/17 mM C/P - 8 M gndHCl buffer, pH 5, as follows: For 1 μM RBH, dilute 10 μl 0.1 mM RBH with 990 μl of same buffer; for 100 nM RBH, dilute 50 μl 1 μM RBH with 450 μl buffer; for 75 nM RBH, dilute 300 μl 100 nM RBH with 100 μl buffer; for 50 nM RBH, dilute 200 μl 100 nM RBH with 200 μl buffer; for 25 nM RBH, dilute 200 μl 50 nM RBH with 200 μl buffer; for 10 nM RBH, dilute 50 μl 100 nM RBH with 450 μl buffer; for 5 nM RBH, dilute 200 μl 10 nM RBH with 200 μl buffer; for 1 nM RBH dilute 50 μl 10 nM RBH with 450 μl buffer. Measure the FU of 0.3 ml of each RBH solution at ex/em 560/585 nm (with the spectrofluorometer Shimadzu model RF-1501 set at 10 nm width slit and high sensitivity), using as reagent blank the 33/17 mM C/P - 8 M gndHCl buffer, pH 5. Then, plot the linear RBH standard curve as FU vs pmoles RBH (present in its e.g. 0.3-ml-solutions, which is the same volume that is used for the measurement of the fluorescence of the protein carbonyl-RBH hydrazone; **Suppl. Fig. 3C**).

***Standard curve with BSA_ox_ carbonyls***: Apply the RBH assay on a concentration series 0-100 nM of BSA_ox_ carbonyl solutions made by dilution (with ddH_2_O) of the 23 nmole carbonyl mg^-1^ BSA_ox_ stock solution, prepared as in *Section I*. Measure the FU of the resulting BSA_ox_ carbonyl-RBH hydrazone solutions (made in final 0.3 ml 33/17 mM C/P - 8 M gndHCl buffer, pH 5) at ex/em 560/585 nm (as in Titration A *Procedure*), using also the 33/17 mM C/P - 8 M gndHCl buffer, pH 5, as reagent blank. Then, make a standard curve plot of FU vs pmoles BSA_ox_ carbonyls (**Suppl. Fig. 3C**).

**Part II.** **Test of DNA interference on the RBH assay**

***Reagent setup***

- **1.85 mg (4.281 µmoles carbonyls) ml^-1^ DNA stock solution:** Same as previously reported **[1]**.
- **Reagents of the RBH assay:** As in article’s article’s *Part A. Protocol of the RBH assay*.

***Procedure*:** Sample, S, and SB are prepared in microcentrifuge tubes as 0.14 ml solutions of various DNA quantities (up to 40 µg, by dilution of the 1.85 mg ml^-1^ DNA stock) in final 50 mM NaOH. Then, S and SB are subjected to **steps 1** and **2** of the article’s *Part A. Protocol of the RBH assay*, resulting in the DOC-TCA-acetone-washed S- and SB-DNA pellets, on which the following alternative *Sub-procedures 1* and *2* (data are shown in **Table 2**):

1. *Sub-procedure* *1*. The DOC-TCA-acetone-washed S and SB DNA pellets are dissolved in 0.3 ml 33/17 mM C/P - 8 M gndHCl buffer, pH 5, by vigorous vortexing (as described in **step 4** of the article’s *Part A. Protocol of the RBH assay*). However, the DNA pellets are not solubilized as judged by the zero absorbance of their solubilizate (carefully collected by placing the pipette tip on the opposite to the centrifugal force bottom side of the tube) at 260 nm, compared to control solutions of known quantities of DNA in 33/17 mM C/P - 8 M gndHCl buffer, pH 5. These solutions are prepared by initially dissolving DNA in 20 μl 33/17 mM C/P - 8 M gndHCl buffer (pH 5), which is then alkalinized by mixing with 1 μl 5.3 Μ ΝaΟΗ, and then sequentially diluted to final 300 μl by addition (and vortexing each time) of 50 and 230 μl 33/17 mM C/P - 8 M gndHCl buffer (pH 5), and finally are mixed with 1 μl 2.8 M HCl to adjust the C/P - gndHCl solubilization buffer pH to 5. This result suggests no direct reaction of RBH with DNA. Therefore, insolubility of the DNA pellet in the solubilizing C/P-gndHCl buffer of the RBH assay is the main factor for the non-interference of this assay on protein samples containing DNA. Another factor (verified by the following *Sub-procedure 2*) is the ~100% effective acetone-wash of free RBH that may be non-specifically bound to DNA (this could have been a problem in protein samples contaminated with DNA, where minor quantities of DNA co-solubilized with the proteins in the 33/17 mM C/P - 8 M gndHCl buffer, pH 5).

2. *Sub-procedure* *2*. To quantify RBH moles still possibly non-specifically bound to the DOC-TCA-acetone-washed S- and SB-DNA pellets (thus, to quantify the interference of precipitated DNA on the RBH assay), DNA pellets are again initially dissolved (by repeated pipette suctions) in 20 μl 33/17 mM C/P - 8 M gndHCl buffer (pH 5), which is alkalinized with 1 μl 5.3 Μ ΝaΟΗ, and then diluted to final 300 μl by sequential addition (and vortexing each time) of 50 and 230 μl 33/17 mM C/P - 8 M gndHCl buffer (pH 5), the pH of which is adjusted to 5 by addition of 1 μl 2.8 M HCl. Subsequently, the net FU of the so resulting S and SB solubilizates is measured and converted to nmoles carbonyls (as in **step 4** of the article’s *Part A. Protocol of the RBH assay*), and, then, the DNA concentration in the resulting S and SB solubilizates is quantified via absorbance at 260 nm (from a DNA standard curve made with solutions of known quantities of DNA in 33/17 mM C/P - 8 M gndHCl buffer, pH 5), and normalized per 1 mg DNA.

**Part III. Test of the RBH assay on carbonylated polysaccharides**

The RBH assay was cross-tested on the following CMCellu-based control solutions prepared at maximum concentration 4 mg ml^-1^ as previously shown **[1]**: CMCellu artificially carbonylated by NaClO (designated as CMCellu_NaClO-carbnylated_), CMCellu_NaClO-carbnylated_ decarbonylated by NaBH_4_ (CMCellu_NaBH4-decarbonylated_), CMCellu treated as for CMCellu_NaClO-carbnylated_ but with the omission of the NaClO treatment step (CMCellu_NaClO treatment omitted_), using CMCellu as overall control solution. From each of these stock solutions, 140 µl samples (S) were prepared at three different polysaccharide concentrations (by dilution with ddH_2_O) up to maximum 0.2 mg (corresponding to 0.1 mg glucose equivalents). These, and three 140 µl polysaccharide dilutions to be used as SB were mixed each with 50 μl 132/68 mM C/P - 0.4 M gndHCl buffer (pH 3), 5 μl 1.4 M HCl and 5 μl 1 mM RBH for the S and 5 µl absolute EtOH for the SB, followed by incubation for 1, 2 and 3 hrs in the dark at 37^o^C. The carbonylated polysaccharide in the assay reaction mixtures S and SB was precipitated by 0.6 ml 100% ice-cold acetone after brief vortexing, and centrifugation at 16,000 *g* for 5 min at 4^ο^C. The S and SB precipitates were 2x washed with 0.5 ml 100% ice-cold acetone and vacuum-dried, and the pellet was solubilized in 60 µl 33/17 mM C/P - 8 M gndHCl buffer, pH 5, 10 µl of which were withdrawn for the quantification of the recovered precipitated polysaccharide as glucose equivalents performed as previously shown **[1]**. The remaining 50 µl were mixed with 250 µl 33/17 mM C/P - 8 M gndHCl buffer, pH 5, and the FU of S was measured against that of SB, and expressed per mg polysaccharide or glucose equivalents as already stated in the RBH assay.


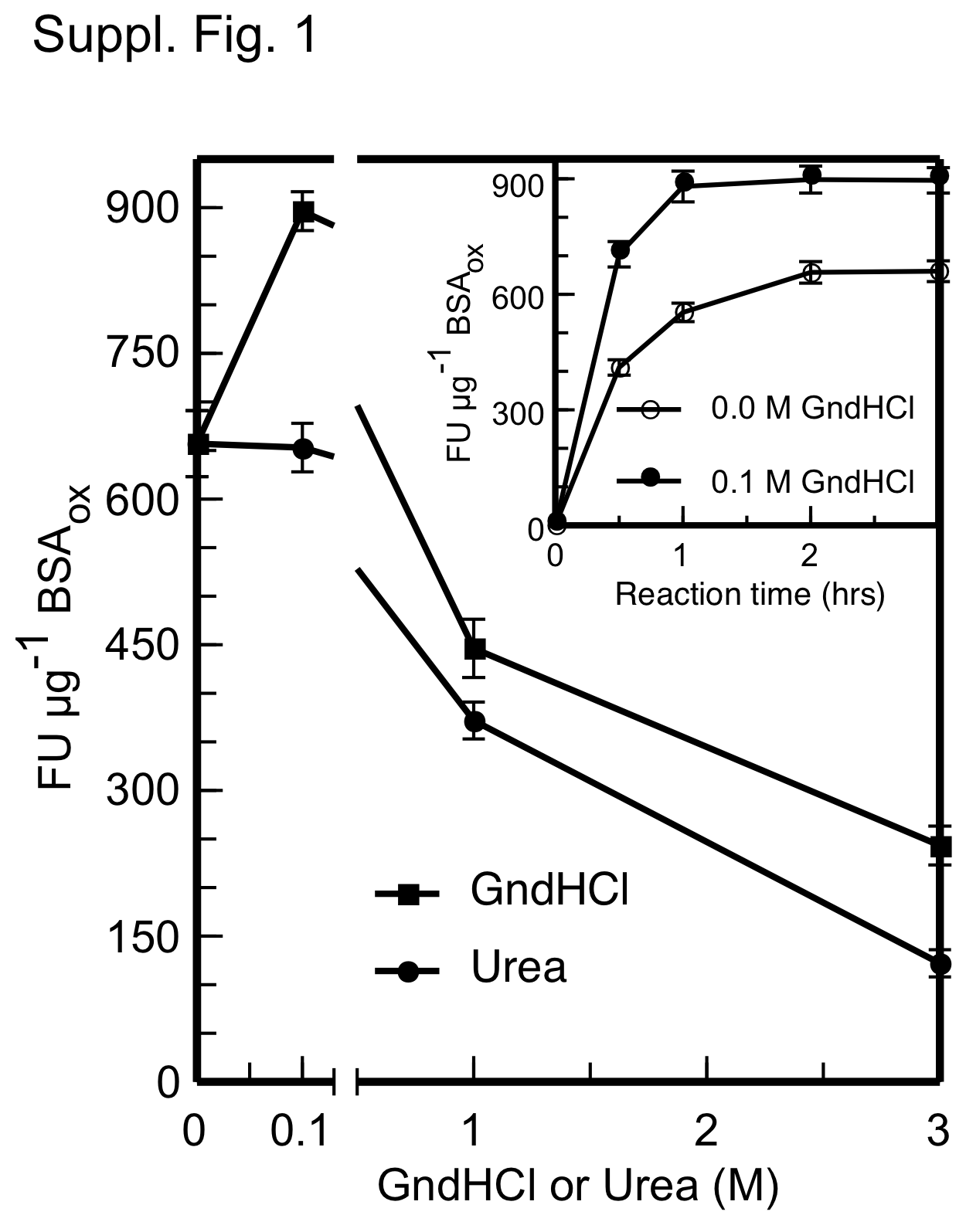
**Figures and legends**

**Suppl. Fig. 1**. Effect of gndHCl and urea on the RBH assay in relation to reaction time in the presence of protein denaturants. Effect of gndHCl and urea on the maximum formation of the protein (BSA_ox_) carbonyl-RBH hydrazone, expressed in fluorescence units (FU) µg^-1^, designated as fluorescence specific activity (FSA). Inset shows the effect of gndHCl and urea on the assay reaction time (and the FSA stability of the hydrazone over time; data not shown) in ±0.1 M gndHCl, the optimum denaturant selected based on the data in the main plot. Similar data (not shown) are also obtained with pepsin_ox_ and lysozyme_ox_. Error bars designate SD.


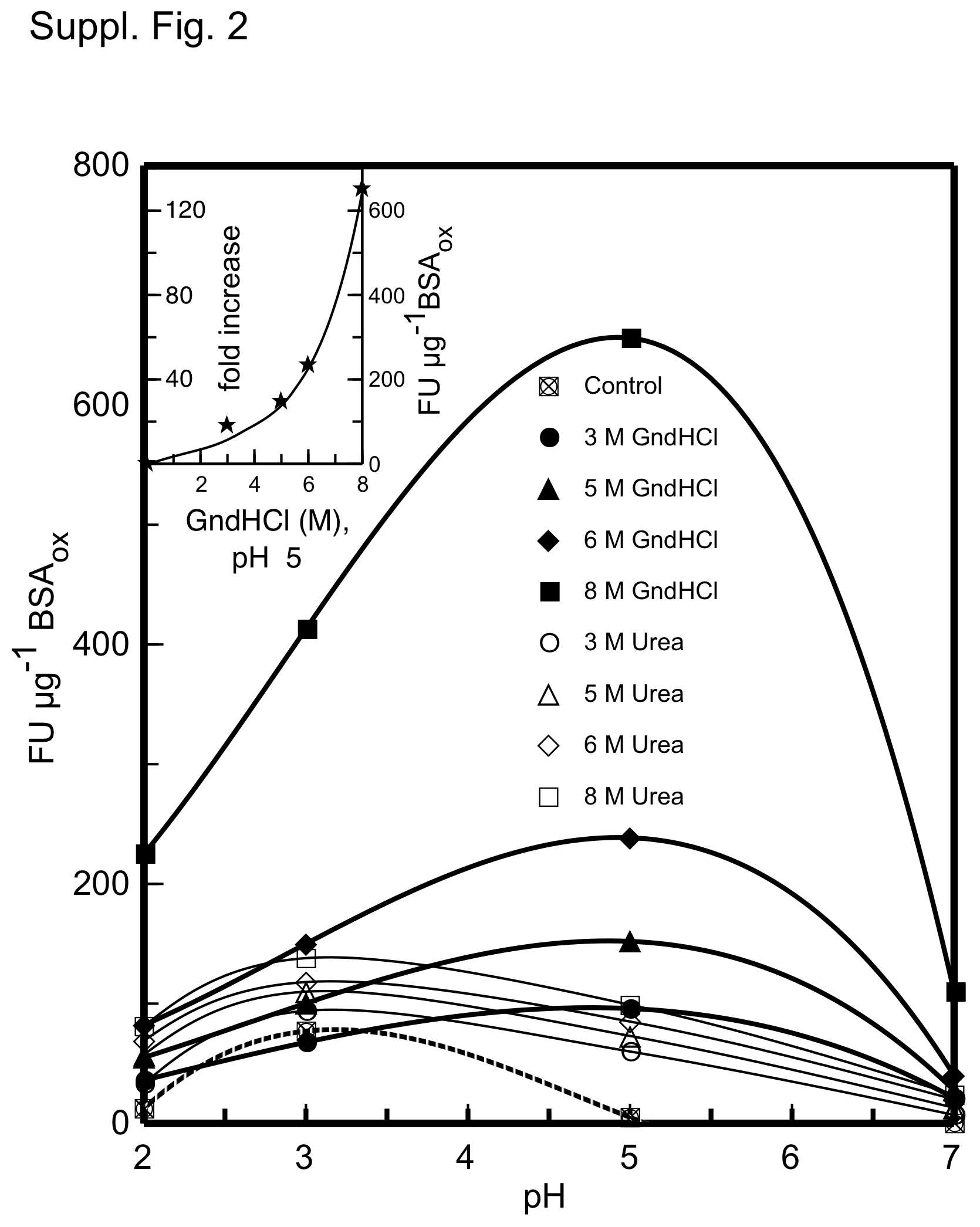


**Suppl. Fig. 2**. Effect of gndHCl and Urea (at various concentrations) vs pH. This is tested on the FSA (FU µg^-1^) of the BSA_ox_ carbonyl-RBH hydrazone against minus gndHCl and urea in the protein solubilization 33/17 mM C/P - gndHCl buffer, pH 3. Inset shows the fold-increase on the FSA of the BSA_ox_ carbonyl-RBH hydrazone at the established (in the main plot) optimum pH 5 as a function of gndHCl concentration in the 33/17 mM C/P buffer. Note that the fold value at 8 M gndHCl can increase ~3 times at saturated gndHCl concentration in the 33/17 mM C/P buffer, pH 5 (data not shown). Similar data (not shown) are also obtained with pepsin_ox_ and lysozyme_ox_.


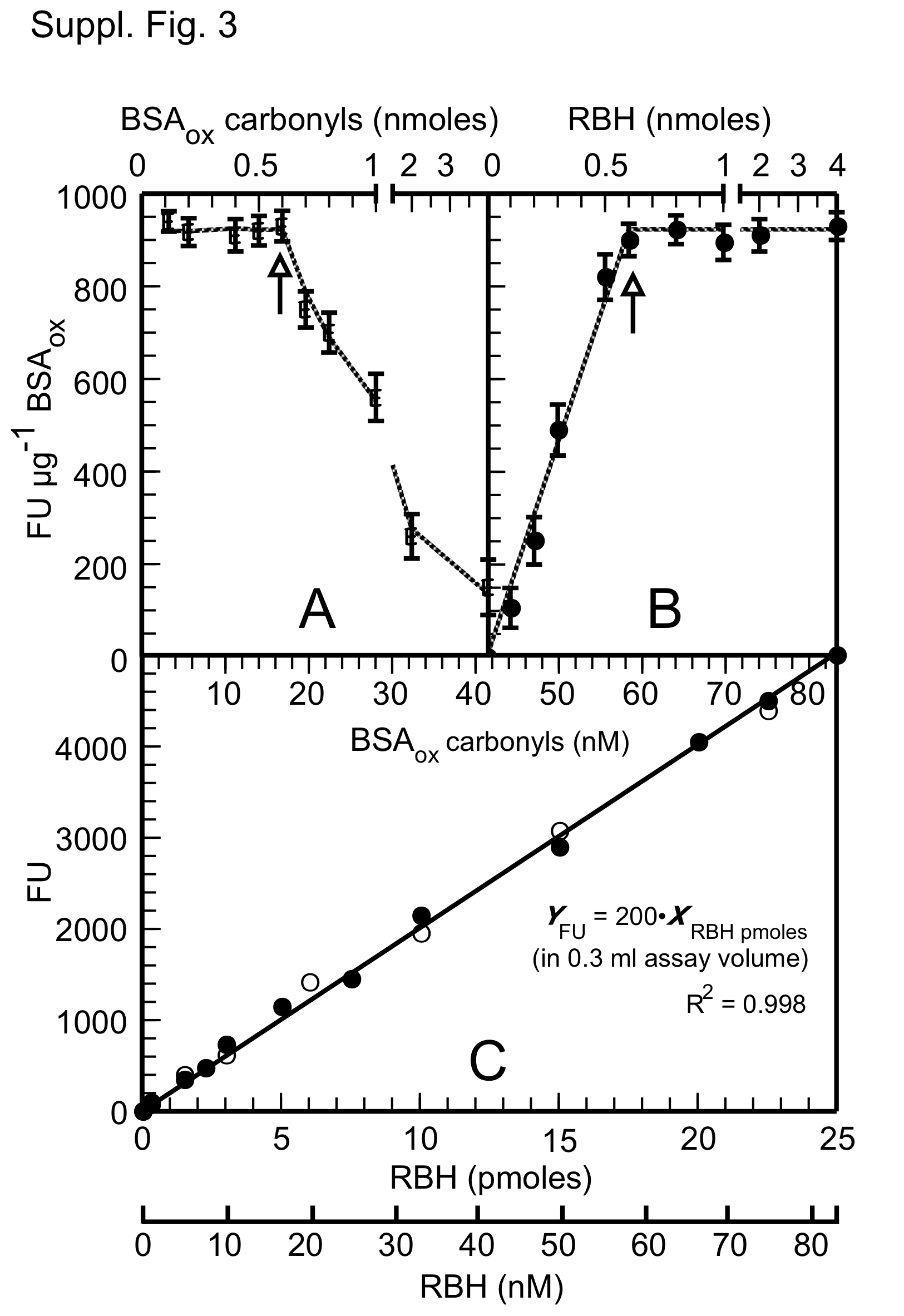
**Suppl. Fig. 3**. Molar stoichiometry of the reaction of RBH with BSA_ox_ carbonyls (same data were obtained with pepsin_ox_ and lysozyme_ox_), and of the molar fluorescence of the protein hydrazone with that of free RBH. Stoichiometry is determined by two independent titratiometry approaches: (A) by the reaction of an RBH constant quantity (0.6 nmoles) with various nmoles BSA_ox_ carbonyls (open circles); (B) by the reaction of a constant quantity (0.6 nmoles) of BSA_ox_ carbonyls with various nmoles RBH (filled circles). Dotted lines in plots **A** and **B** are the theoretical values of the BSA_ox_ carbonyl-RBH hydrazone FSA (FU µg^-1^ BSA_ox_), obtained by the identical standard curves of RBH and BSA_ox_ carbonyls (filled and open circles, respectively, in plot **C**; their construction follows), which show that the molar stoichiometric fluorescence ratio of carbonyl-RBH hydrazone and free RBH is 1:1 (using same solubilization buffer at 8 M gndHCl and pH 5). Curve data shown in plots **A** and **B** by the circles represent the actual BSA_ox_ carbonyl-RBH hydrazone experimental FSA values, and they fit very well to the corresponding theoretical curves. Both arrows in plots A and B show the formation of BSA_ox_ carbonyl-RBH hydrazone at equimolar 1:1 ratio of BSA_ox_ carbonyls and RBH. (C). Depicted standard curve is actually the average of one standard curve made by various concentrations of RBH (filled circles), and the second standard curve made by equimolar (to RBH) concentrations of BSA_ox_ protein carbonyls (open circles). The slopes of both standard curves are identical and verify again that the molar stoichiometric fluorescence ratio of the protein carbonyl-RBH hydrazone and free RBH is 1:1. Note that at 8 M gndHCl in the 33/17 mM C/P buffer, pH 5 (the near maximum solubility of gndHCl at RT), the slope is ~200 FU per RBH pmole (**Suppl. Fig. 3C**), which can even exceed 600 using buffer saturated with gndHCl (data not shown). Error bars designate SD.

**References**

[1] Georgiou, C. D.; Zisimopoulos, D.; Argyropoulou, V.; Kalaitzopoulou, E.; Salachas, G.; Grune, T. Protein and cell wall polysaccharide carbonyl determination by a neutral pH 2,4-dinitrophenylhydrazine-based photometric assay. *Redox Biology,* DOI: https://doi.org/10.1016/j.redox.2018.04.010 2018.

[2] Li, X.; Duerkop, A.; Wolfbeis, O. S. A fluorescent probe for diacetyl detection. *J. Fluoresc.* **19:** 601-606; 2009.

[3] Arnold, U.; Ulbrich-Hofmann, R. Quantitative protein precipitation from guanidine hydrochloride-containing solutions by sodium deoxycholate/trichloroacetic acid. *Anal. Biochem.* **271:** 197-199; 1999.

[4] Bensadoun, A.; Weinstein, D. Assay of proteins in the presence of interfering materials. *Anal. Biochem.* **70:** 241-250; 1976.

[5] Georgiou, C. D.; Grintzalis, K.; Zervoudakis, G.; Papapostolou, I. Mechanism of Coomassie brilliant blue G-250 binding to proteins: a hydrophobic assay for nanogram quantities of proteins. *Anal. Bioanal. Chem.* **391:** 391-403; 2008.
